# Combinatorial analysis of lupulin gland transcription factors from R2R3Myb, bHLH and WDR families indicates a complex regulation of *chs*_H1 genes essential for prenylflavonoid biosynthesis in hop (*Humulus Lupulus *L.)

**DOI:** 10.1186/1471-2229-12-27

**Published:** 2012-02-20

**Authors:** Jaroslav Matoušek, Tomáš Kocábek, Josef Patzak, Zoltán Füssy, Jitka Procházková, Arne Heyerick

**Affiliations:** 1 Biology Centre ASCR v.v.i, Institute of Plant Molecular Biology, Branišovská 31, 370 05 České Budějovice, Czech Republic; 2 Hop Research Institute, Co. Ltd, Kadaňská 2525, 438 46 Žatec, Czech Republic; 3 Faculty of Science, University of South Bohemia, Branišovská 31, 370 05 České Budějovice, Czech Republic; 4 Laboratory of Pharmacognosy and Phytochemistry, Faculty of Pharmaceutical Sciences, Ghent University, Harelbekestraat 72, B-9000 Ghent, Belgium

## Abstract

**Background:**

Lupulin glands of hop produce a specific metabolome including hop bitter acids valuable for the brewing process and prenylflavonoids with promising health-beneficial activities. The detailed analysis of the transcription factor (TF)-mediated regulation of the oligofamily of one of the key enzymes, i.e., chalcone synthase CHS_H1 that efficiently catalyzes the production of naringenin chalcone, a direct precursor of prenylflavonoids in hop, constitutes an important part of the dissection of the biosynthetic pathways leading to the accumulation of these compounds.

**Results:**

Homologues of flavonoid-regulating TFs *HlMyb2 *(M2), *HlbHLH2 *(B2) and *HlWDR1 *(W1) from hop were cloned using a lupulin gland-specific cDNA library from the hop variety Osvald's 72. Using a "combinatorial" transient GUS expression system it was shown that these unique lupulin-gland-associated TFs significantly activated the promoter (P) of *chs*_H1 in ternary combinations of B2, W1 and either M2 or the previously characterized *Hl*Myb3 (M3). The promoter activation was strongly dependent on the Myb-P binding box TCCTACC having a core sequence CCWACC positioned on its 5' end region and it seems that the complexity of the promoter plays an important role. M2B2W1-mediated activation significantly exceeded the strength of expression of native *chs*_H1 gene driven by the 35S promoter of CaMV, while M3B2W1 resulted in 30% of the 35S:*chs*_H1 expression level, as quantified by real-time PCR. Another newly cloned hop TF, *Hl*Myb7, containing a transcriptional repressor-like motif pdLNLD/ELxiG/S (PDLNLELRIS), was identified as an efficient inhibitor of *chs*_H1-activating TFs. Comparative analyses of hop and *A. thaliana *TFs revealed a complex activation of P*chs*_H1 and P*chs*4 in combinatorial or independent manners.

**Conclusions:**

This study on the sequences and functions of various lupulin gland-specific transcription factors provides insight into the complex character of the regulation of the *chs*_H1 gene that depends on variable activation by combinations of R2R3Myb, bHLH and WDR TF homologues and inhibition by a Myb repressor.

## Background

Hop (*Humulus lupulus *L.) plants are mainly cultivated for the brewing industry, as a source of flavor-active secondary metabolites contained in the lupulin glands, i.e. glandular trichomes that develop in the hop female inflorescences (cones). In addition, hop has been known for a long time in traditional medicine and recently several compounds in the lupulin metabolome, including hop bitter acids and prenylated flavonoids, have received particular attention in view of their highly interesting medicinal properties e.g., [1-3]. Xanthohumol (X), the principal prenylated chalcone in the lupulin glands is a fascinating cancer-chemopreventive compound exhibiting a broad spectrum of inhibition mechanisms at all stages of carcinogenesis [4]. Although X was shown to be poorly bioavailable [2,5-7], interesting anti-inflammatory in vivo results have been obtained in specific target tissues such as the liver [8]. Another lupulin-derived prenylflavonoid, 8-prenylnaringenin (8-PN), is one of the most potent phytoestrogens known to date [9,10]. Together with its precursor isoxanthohumol which can be metabolized in the body by the gut microbiota to 8-PN [11], these compounds are indicated as the active ingredients of hop extracts targeting relief of menopausal symptoms [12,13].

The biosynthesis of naringenin chalcone in hop cones as a prenylflavonoid precursor is attributed to the gene encoding the CHS_H1 protein having a so-called "true" chalcone synthase (EC 2.3.1.74) activity, by which it efficiently catalyzes the production of naringenin chalcone by condensation of three malonyl-CoA units and *p*-coumaroyl-CoA [14,15]. Moreover, recombinant CHS_H1 can utilize isovaleryl and isobutyryl CoA substrates, albeit at a low rate, and, therefore, could also be involved in the biosynthesis of hop bitter acids [15]. CHS_H1 is encoded by an oligofamily of genes having very specific expression in hop cones [16]. Other chalcone synthase-like enzymes associated with lupulin glands have been described. For instance, valerophenone synthase (VPS) [17] is an enzyme which may be considered as a major component in the biosynthesis of hop bitter acids. The VPS-like homologue CHS4 also shows high expression in lupulin glands, however the enzyme does not catalyze the formation of naringenin chalcone and its function is currently still unknown [15,18].

The complexity of the promoter elements of the *chs*_H1 genes suggests the involvement of several types of transcription factors (TFs), mainly from Myb, bHLH, and bZip families [16,19-21], in either independent or combinatorial pathways [22]. Independent or combinatorial activity of TFs in the regulation of flavonoid biosynthetic pathways has been described in several recent reviews [23-26]. Three TFs, i.e., R2R3Myb (M), bHLH (B), and WDR (W), exert combinatorial activation by formation of ternary complexes (MBW complexes) through protein:protein interactions [26]. Such MBW complexes are highly organized and each subunit fulfills a specific function such as binding to DNA, activation of expression of a target gene or stabilization of the transcription factor complex [23]. MBW complexes have been clearly identified in the flavonoid biosynthetic pathway of *Arabidopsis thaliana *and *Petunia hybrida*, respectively, as a TT2/TT8/TTG1 complex (Transparent Testa 2/Transparent Testa 8/Transparent Testa Glabra 1) driving coloration of the seed coat [27,28] and a triple combination complex AN2/AN1/AN11 (Anthocyanin 2/1/11) regulating anthocyanin accumulation in the corolla; for reviews, see also [23,26,29]. The regulation of the flavonoid biosynthesis pathway by ternary complexes has also been shown in peas [30] and *Lotus japonicus *[31] and has been proposed for *Perilla frutescens *[32], Japanese morning glory [33], grapevine [34] and *Pyrus *[35].

Besides triple combinations, numerous examples from classic genetic or molecular genetic studies using ectopic TF expression analysis have documented or predicted enhanced flavonoid biosynthesis by binary complexes composed of two TFs from the R2R3Myb, bHLH, and WDR classes [34,36-40].

For the hop *chs*_H1 genes, direct promoter activation by the heterologous PAP1 TF from *Arabidopsis thaliana *and *Hl*bZIP1 and *Hl*bZIP2 TFs from hop has been demonstrated previously using a transient expression system [16,21]. Two hop R2R3Myb TFs, i.e. *Hl*Myb1 and *Hl*Myb3, have been characterized in our previous studies [19,20] and diverse biological effects caused by *Hl*Myb3 subvariants have been demonstrated in heterologous transgenotes [20]. Although these Myb TFs were suggested to be involved in the lupulin metabolome production based on sequence similarity and specific expression in hop cones [19,20], their influence on the activation of *chs *genes has not been investigated in detail.

In the present work, we cloned novel lupulin-specific TFs and showed by a combinatorial transient expression assay that the *Hl*bHLH2 and *Hl*WDR1 TFs strongly activate the *chs*_H1 genes in combinations with *Hl*Myb2 and *Hl*Myb3 TFs from hop, suggesting the formation of ternary complexes. This study confirms that the mode of action of the TFs strongly depends on the composition of the *chs *promoter and that *Hl*Myb7 acts as a repressor of activating complexes.

## Results

### Cloning, comparative sequence and genomic analyses of R2R3Myb, bHLH and WDR transcription factors from hop

During our previous work, we cloned two authentic R2R3Myb TFs having specific expression in cones of the Czech hop variety Osvald's 72, i.e., *Hl*Myb1 [GenBank:AJ876882] [19] and *Hl*Myb3 [GenBank:AM501509] [20]. In the present work, we isolated and purified lupulin glands from Osvald's 72 according to Nagel et al. [41] and constructed a lupulin gland-specific cDNA library. This cDNA library is more specific and more complex (3.5 × 10^6 ^pfu) than the previously constructed cDNA library from hop inflorescences and cones [19]. We used sequence motifs for two R2R3Myb TFs available in the GenBank database [no. AB292245 and AB292244], to amplify *Hl*Myb2 [GenBank: FN646081] and *Hl*Myb7 [GenBank:FR873650], respectively, from the cDNA library. Both clones differed in several point mutations and, in addition, an 18 bp deletion was found for the clone *Hl*Myb2 in comparison with the original database sequence [GenBank:AB292245]. The full-length clones of HlbHLH2 [GenBank:FR751553] were obtained by screening of hop EST sequences after pre-selections of 5' and 3' cDNA motifs in the TrichOME database (http://trichome.noble.org/trichomedb/). To get full length cDNA clones of HlWDR1 [GenBank: FN689721] we firstly identified the 5' region from EST of the *H. lupulus *var. Phoenix. Full-length cDNA was obtained from a lupulin gland-specific library of the Osvald's 72 cv. using a combination of a 5'primer and an oligo-dT anchor primer followed by nested PCR as described in Methods. The authenticity of all newly obtained cds sequences was verified by sequencing of the high-fidelity RT-PCR clones.

**Figure 1:**
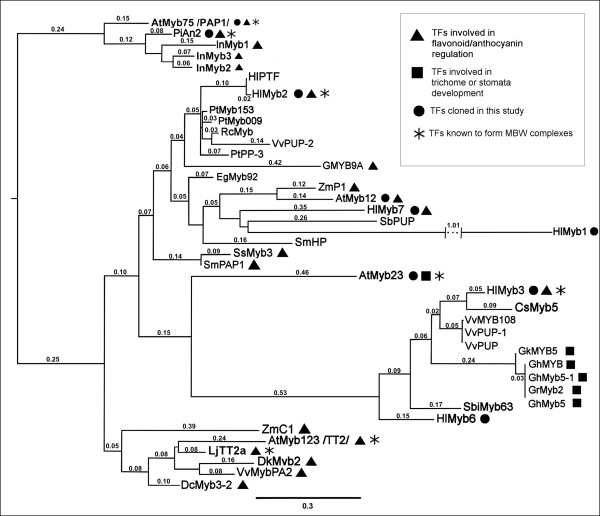
**Phylogenetic analysis for amino acid sequences of selected plant MYB TFs**. The sequences of R2 and R3 domains of 40 selected plant MYB TFs were compared with PhyML v.3.0 and the tree was rooted along *At*Myb075 using FigTree v.1.3.1. (see Methods). The lengths of interrupted branches are expressed in aLTR values corresponding to the scale. See additional file 1[Supplementary-material S2] for amino acid alignment, ID numbers and annotations of individual TFs.

At present, 7 full-length R2R3Myb factors from the Osvald's 72 cultivar are available in the sequence database. From these sequences, the two newly cloned *Hl*Myb2 and *Hl*Myb7 showed high amino acid identity from 67 to 82% within the R2R3 domain to *A. thaliana *AtMyb12 [GenBank: NM_130314] and P1 TFs from *Z. mays *[GenBank: NM_001111873] involved in the flavonoid biosynthesis. A more wide comparison with various related R2R3 Myb TFs selected from BLAST analysis shows the positioning of *Hl*Myb7 and *Hl*Myb2 in different closely related clusters (Figure 1). The previously cloned *Hl*Myb1 [19] is clustering together with *Hl*Myb7, however, this sequence is less related to ZmP1. *Hl*Myb3 that was found earlier as metabolome regulator [20] is clustering together with *C. sativa, V. vinifera*, and *G. hirsutum *Mybs as described previously [20]. This TF is clustering together with *Hl*Myb6, while two other hop Mybs 4 and 5 are quite unrelated and are not included in the analyzed dataset of Myb sequences (see Additional file 1). Within the presented R2R3Mybs, some of them were proven to be involved in anthocyanin metabolism or/and trichome development forming ternary BMW complexes such as AtTT2 [GenBank: NM_122946], AtMyb75 [GenBank: NM_104541], AtMyb23 [GenBank: NM_123397] and PiAN2 from *P. integrifolia *[GenBank: AF146703].

*Hl*Myb2 represents a typical R2R3Myb, with a predicted mass of 29.7 kDa and a pI of 8.16. *Hl*Myb7 is an R2R3Myb with a predicted protein mass of 29.6 kDa and pI of 8.19. These characteristics are close to other Myb TFs forming ternary complexes like TT2 from *A. thaliana *(29.6 kDa, pI 8.8) or AN2 from *P. hybrida *(29.0 kDa, pI 7.23). In the same range of the molecular mass, but more acidic, are the subvariants of hop Myb3, i.e., s-*Hl*Myb3 (29.9 kDa, pI 6.4) and l-*Hl*Myb3 (30.3 kDa, pI 6.19). In the C-terminal region of *Hl*Myb7, we identified a PDLNLELRIS motif which conforms to the consensus pdLNLD/ELxiG/S characteristic for plant Myb repressors [42] described in *A. thaliana *and *Z. mays *[43,44] and showing similarities to II AP2/ERF transcriptional repressors [45]. While the R3 repeat in *Hl*Myb2 perfectly matches the sub-group IIIf bHLH interaction motif [DE] L×2[RK]×3L×6L×3R [24,38,46], the *Hl*Myb7 sequence matches this motif only partly (66% identity) and in *Hl*Myb3 this motif is absent (see Additional file 2). However, the following consensus sequence. LllcLHphhGNRWShIAth (according to Bork consensus, http://coot.embl.de/Alignment//consensus.html) is characteristic for this interacting region, where most positions of putative interacting residues (underlined) are hydrophobic amino acids exposed on the surface.

**Figure 2:**
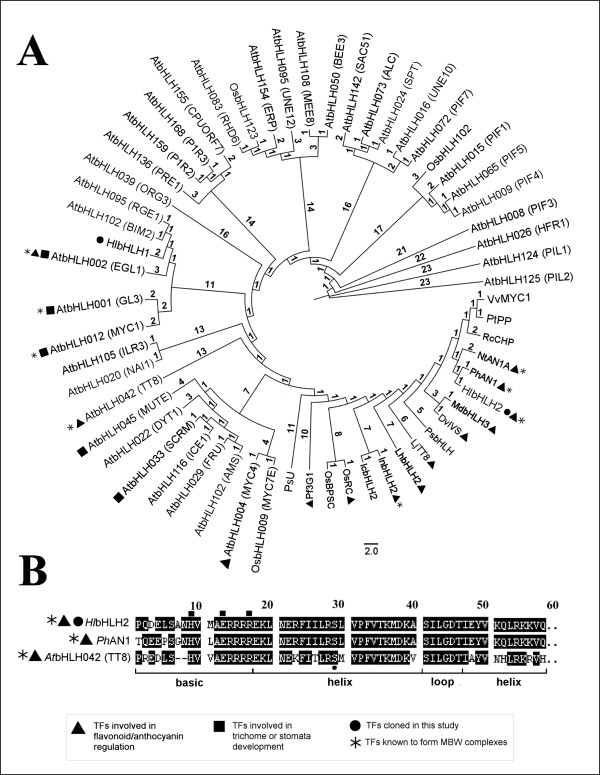
**Phylogenetic analysis for amino acid sequences of selected plant bHLH TFs (A) and Alignment analysis of the bHLH2 domain (B)**. The sequences of bHLH domain of 58 selected plant bHLH TFs were analyzed using PhyML v. 3.0. The polar tree cladogram presented in panel A was visualized using FigTree v.1.3.1. (see Methods). The lengths of branches are expressed in aLTR values corresponding to the scale. Alignment of the bHLH domains of hop *Hl*bHLH2, petunia PhAN1 and *A. thaliana *AtbHLH042 (TT8) is shown in panel B. The bHLH domain of *Hl*bHLH2 was identified by similarity to the TT8 domain as described by [48]. Amino acids within the basic region crucial for G-box DNA binders [47] are indicated by squares. See additional file 3 for amino acid alignment, ID numbers and annotations of individual TFs.

The cloned *HlbHLH2 *cDNA sequence encoded for an acidic (pI 5.1) protein having a molecular weight of 77.1 kDa. According to analysis of bHLH domains of selected plant bHLH TFs sequences from BLAST and from the dataset of Carretero-Paulet et al.[47], *Hl*bHLH2 shows the highest similarity to bHLH AN1 from *P. hybrida *(90% amino acid identity within the bHLH domain). It is clustering with several bHLH TFs that are involved in the regulation of the anthocyanin pathway, and belongs to wide cluster covering TFs with biological functions connected to flavonoid/anthocyanin metabolism, trichome, stomatal complex and flower development within functional classes 1, 2, 5, and 10 as arranged by Carretero-Paulet et al.[47] (Figure 2A). Some of the related sequences were described to form MBW complexes (Figure 2A, more in detail described in Additional file 3). Comparisons of the bHLH basic regions revealed the presence of amino acid motif (H9, E13, R17) in the *Hl*bHLH2 domain characteristic for G-box binders [47], similarly to well characterized bHLH AN1 from *P. hybrida *and bHLH TT8 from *A. thaliana *[48] (Figure 2B).

**Figure 3:**
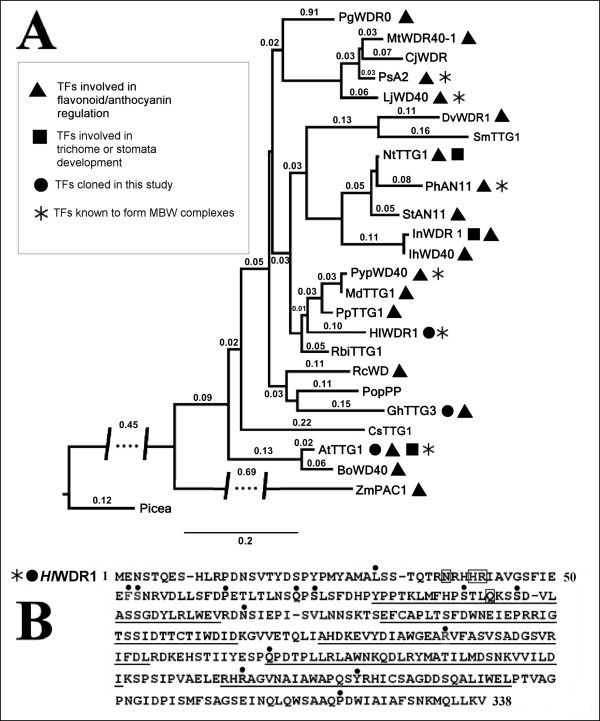
**Phylogenetic analysis for amino acid sequences of selected plant WDR TFs (A) and comparison with the predicted *Hl*WDR1 sequence (B)**. Twenty five selected entries of plant WDR TFs (for the data set see additional file 4) were analyzed using PhyML v. 3.0 and the trees were visualized using FigTree v.1.3.1. (see Methods). The tree has been rooted using the single representative from *Picea sitchensis*. The lengths of the interrupted branches are expressed in aLTR values corresponding to the scale. The predicted *Hl*WDR1 protein sequence is shown with differences indicated in comparison with AN11 protein from petunia with the differences indicated. Non-equivalent amino acids to *Ph*AN11 are designated by dots, gaps are designated by hyphens and amino acid insertions are boxed. WD repeats identified by analogy to PhAN11 [49] are underlined.

The cloned *HlWDR1 *cDNA encodes for a 38 kDa protein having a pI of 4.9. Phylogenetic analysis of this TF using sequences selected from BLAST revealed closely related WDR clusters within the group of dicotyledonous plants (Figure 3A, for more details see Additional file 4). Most of these TFs were proven to regulate anthocyanin metabolism and some of them to form MBW complexes. *Hl*WDR1 showed the highest similarity to recently reported sequences isolated from pear, apple, plum and raspberry (Figure 3A). Hop WDR1 shares 78.6% of identical amino acid with the AN11 protein from *P. hybrida *originally characterized as a plant WDR by de Vetten et al. [49] (Figure 3B). More detailed comparisons of *Hl*WDR1 with *Ph*AN11 identified five polypeptide sequences that constitute the WD-repeats according to [49]. Only nine non-equivalent positions were identified in the *Hl*WDR1 repeats in comparison to *Ph*AN11 (Figure 3B, sequences underlined).

**Figure 4:**
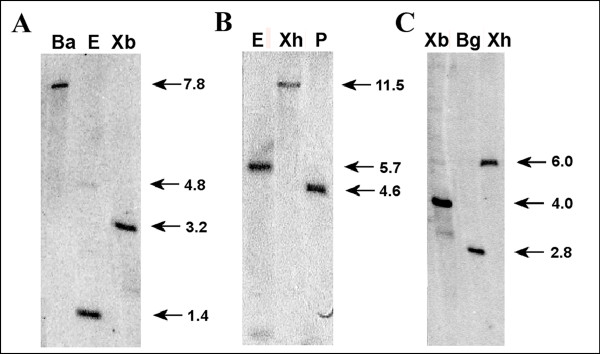
**Southern blot analysis of 5 μg genomic DNA isolated from the hop cv. Osvald's clone 72. The blot was hybridized with a ^32^P-labeled cDNA fragment**. (**A**) *Hl*WDR1 gene: *BamH*I (Ba) and *Xba*I (Xb) do not cut within the *Hl*WD40 gene, *EcoR*I (E) has one restriction site 146 bp from the 5'end of the cDNA. (**B**) *Hl*bHLH2 gene: *EcoR*I (E), *Xho*I (Xh) and *Pst*I (P) do not cut within the *Hl*bHLH2 gene. (**C**) *Hl*Myb2 gene: *Xba*I (Xb) does not cut in the *Hl*Myb2 gene, *Bgl*II (Bg) and *Xho*I (Xh) have one restriction size, but only 69 resp. 43 bp from the 3'end of the cDNA. The arrows show the position of the restriction fragment hybridizing with the cDNA probe fragment. The estimated size is indicated in kilobases (kb).

In order to assess the complexity of the genes encoding the newly cloned hop transcription factors *Hl*Myb2, *Hl*bHLH2 and *Hl*WDR1, Southern blot analysis was carried out on genomic DNA isolated from the hop cultivar Osvald's 72 and digested with several restriction enzymes selected on the basis of the known cDNA sequences. Hybridization with probes created from cDNA fragments of *Hl*Myb2, *Hl*bHLH2 and *Hl*WDR1 genes showed single bands for all tested genes (Figure 4) when digested with *Eco*RI (*HlbHLH2 *and *HlWDR1*), *Xho*I (*HlbHLH2 *and *HlMyb2*), *Xba*I (*HlMyb2 *and *HlWDR1*), *Pst*I (*HlbHLH2*), *Bgl*II (*HlMyb2*) and *Bam*HI (*HlWDR1*) enzymes. This suggests that the cloned hop transcription factors *HlMyb2, HlbHLH2 *and *HlWDR1 *are rather unique in the hop genome of Osvald's 72. The occurrence of two bands in the case of *HlbHLH2 *cleaved with *Eco*RI (5.7 and 0.6 kb) and *HlMyb2 *cleaved with *Xba*I (6.0, 4.0 and 3.2 kb) is likely due to cleavage within some intron(s).

### Comparative expression level analyses of the transcription factors *HlMyb *2/7, *HlbHLH2 *and *HlWDR1 *in hop tissues show specificity for lupulin glands

**Figure 5:**
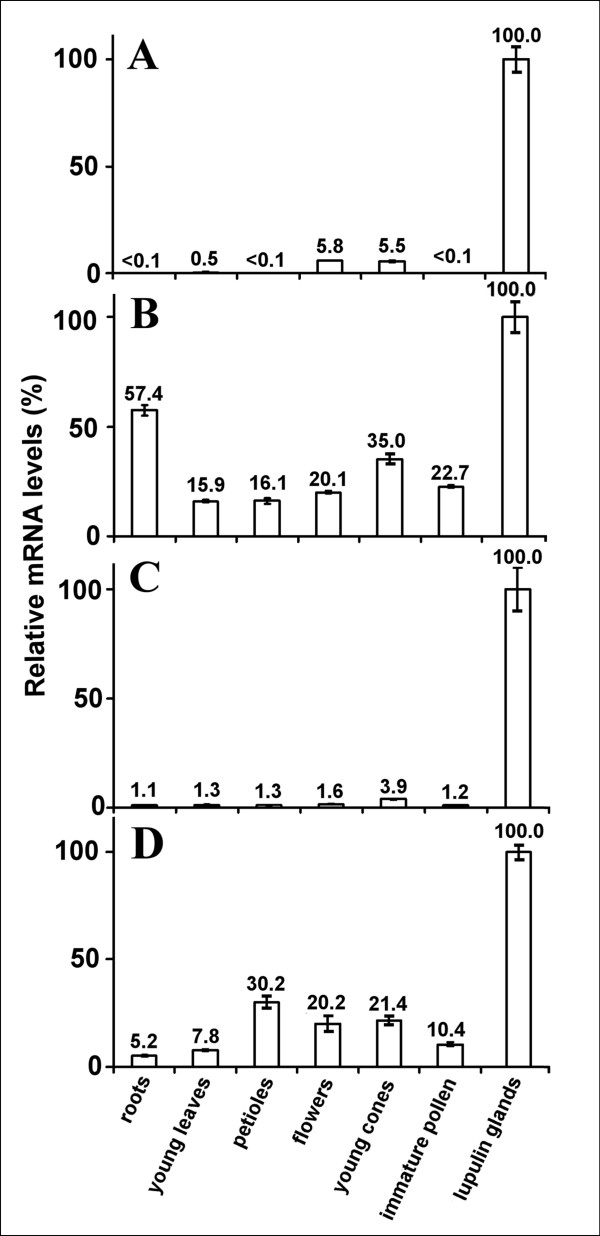
**Real Time RT PCR analysis of hop TFs. *HlMyb2 *(A), *HlMyb7 *(B), *HlbHLH2 *(C) and *HlWDR1 *(D) expression in lupulin glands and other tissues of *H. lupulus *Osvald's clone 72 as designated in the figure**. GAPDH was used as house-keeping gene.

Myb, bHLH and WDR genes from hop were amplified by the use of sequence motifs preselected from trichome databases and cloned from a cDNA library prepared from purified glandular trichomes. This corroborated to an initial specificity for lupulin glands. In order to verify the specificity of the expression of the cloned hop TFs *HlMyb2, HlMyb7, HlbHLH2, HlWDR1 *we analyzed mRNA levels in various tissues using Real-Time quantitative PCR (RT qPCR). Glyceraldehyde-3-phosphate dehydrogenase (GAPDH) mRNA was selected as a housekeeping gene for normalization based on previous experience and reports from others [41,50,51]. In order to avoid RNA contaminations from hop cone bracts, the lupulin glands of clone Osvald's 72 were carefully separated and purified as recommended by Nagel et al. [41]. The GAPDH-normalized results show that *HlMyb2 *expression is highly specific for lupulin glands (Figure 5A) as the relative expression levels in flowers and young cones only amounted to approximately 5% while in other tissues only traces of the PCR product were found. Almost identical results were obtained for *HlbHLH2 *(Figure 5C), where expression of only about 1% were detected in tissues other than purified lupulin glands. A 4% relative expression level was detected in whole young cones. The expression of *HlWDR1 *was less specific for lupulin glands (Figure 5D). A highly significant relative expression level of this TF, reaching more than 20%, was observed in petioles, flowers and in whole young cones, where maturating lupulin glands comprise only a negligible fraction of total weight of the cone tissues. In addition, immature pollen was found to have *HlWDR1 *mRNA relative expression levels of about 10%. Furthermore, lower expression of *HlWDR1 *was detected in somatic tissues such as young leaves and roots (Figure 5D). The lowest specificity for lupulin glands was observed for *HlMyb7*, where roots and whole young cones were found to contain a relative expression level of 57% and 35%, respectively, whereas other tissues including leaves, flowers and immature pollen also showed high expression levels of over 15% (Figure 5B).

### Combinatorial transient expression analysis of selected lupulin gland TFs revealed a complex activation of p*chs*_H1 and *chs*_H1

According to our results, *HlMyb2 *and *HlbHLH2 *exhibited high specific expression in lupulin glands similar to the previously investigated *HlMyb3 *[21]. Although the expression of *HlWDR1 *and *HlMyb7 *showed a lower specificity, their relative expression levels were still most pronounced in the glandular trichomes. Given the high sequence similarity of all these TFs to known regulators of the flavonoid pathway and their specific expression pattern, it is likely that these TFs are involved in the regulation of genes like *chs*_H1, that are anticipated to be involved in the biosynthesis of secondary metabolites in the lupulin glands [52].

**Figure 6:**
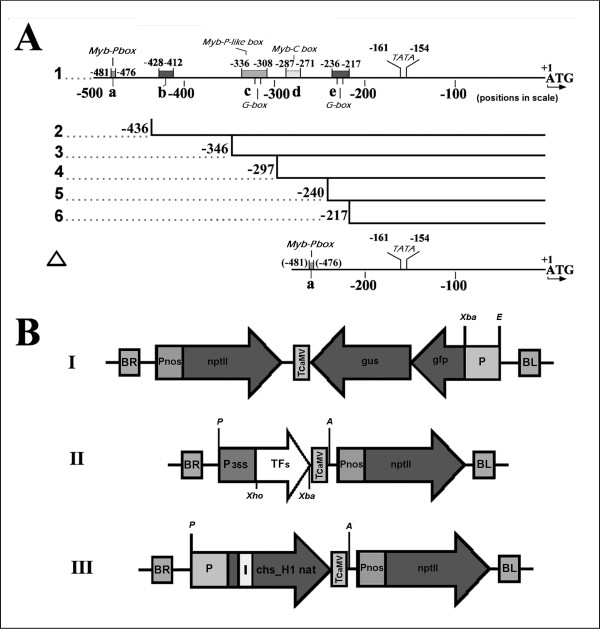
**Schematic drawing of the analyzed variants of the *chs*_H1 promoter (A) and expression cassettes within the T-DNA parts of the plant vectors used for leaf infiltration (B)**. The promoter scheme (**A**) is in scale. I: P*chs*_H1 is summarized as described previously [16] and analyzed in this work. See text for further details.: a, Myb-P box; b, Myb-like box; c, Myb-P/bHLH binding site with G-box; d, plant MYB IIG-like binding site; e/bHLH binding site with box with G-box; The plant vector schemes (**B**) are not in scale. I: general scheme of the vector pBGF-0 containing the analyzed promoters (P). II: the general cassette of vector pLV-07 bearing the 35S-driven transcription factor (TF) from hop. Cassette III shows the native *chs*_H1 gene (vector pLV-67). Coding sequences are in dark gray, promoters are marked by P and the intron is marked by I. BR and BL, right and left T-DNA borders, respectively. NptII designates the neomycin phosphotransferase gene for resistance to kanamycin. This gene is driven by the nopalin synthase promoter (Pnos). Terminators of CaMV (TCaMV) are shown. Restriction sites XbaI (X), EcoRI (E), AscI (A), and PacI (P) were used for the integration promoters and cassettes in the plant vector.

To assess the function of the cloned TFs we have used a"combinatorial transient expression"system that was developed previously [16,21]. In this assay the activation of *chs*_H1 promoter (P*chs*_H1) fused to the GUS reference gene (Figure 6) is measured after co-infiltration of *A. tumefaciens *bearing P*chs*_H1 and hop TFs vectors in various combinations into *N. benthamiana *leaves. Only negligible background signal resulting from activation of P*chs*_H1 by internal leaf TFs was detected [16,21].

P*chs*_H1 contains several Myb and bHLH-binding boxes as described previously [16] and analyzed further in this work (Figure 6A). According to the PLACE database the sequence TCCTACC (MybPZm box) containing the consensus of the *Z. mays *P factor binding box CCWACC [53] was identified at position -481 to -476. This potential TF binding box was of high interest in relation to the significant similarity of the newly cloned *Hl*Myb2 and *Hl*Myb7 to this *Zm*P1 factor (Figure 1). At position -428 to -412 a Myb-like box was identified using the Genomatix database (http://www.genomatix.de) with a consensus for GA-Myb proteins [54]. For position -336 to -328 the sequence CACCAAAC conforming to the Myb-P-related consensus sequence MACCWAMC was found using the PLACE database [55] and at position -287 to -271 a plant MYB IIG-type-like binding site was identified, i.e., TAAGGTAGTTGA (mismatch underlined), which is similar to the *Z. mays *C1 Myb-domain protein (Myb-C box) [56]. In addition, two typical bHLH G-binding boxes with the sequence CACGTG, that could be important for *Hl*bHLH2-mediated activation of P*chs*_H1 [57], were identified at positions -224 to -229 and -313 to -318 (Figure 6A).

The maximum values of transient GUS activity was reached at 66 to 92 h post-infiltration and declined thereafter dependent on the TF or TFs combination co-infiltrated. For example, *Hl*Myb3 variants and all complexes containing the *Hl*Myb3 gene peaked at 4 days post infiltration (dpi), whereas the *Hl*Myb2 gene reached maximum activity 66-68 h dpi, except for the M2W1 binary complex.

**Figure 7:**
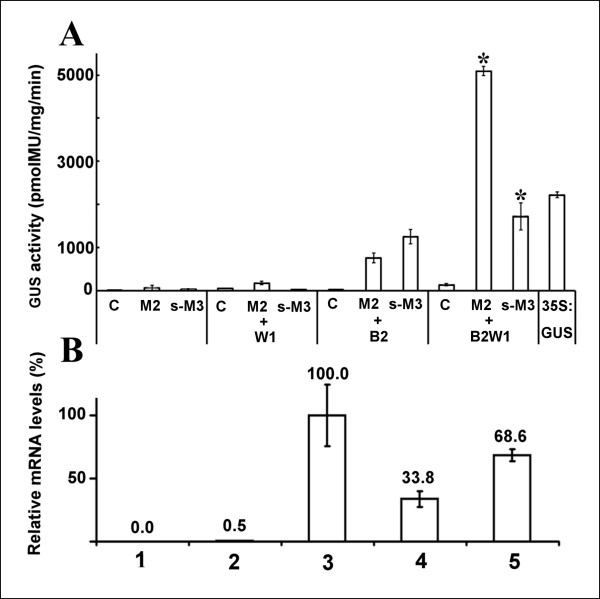
**Analysis of activation of the *chs*_H1 promoter (A) and the whole *chs*_H1 gene (B)**. The combinatorial transient expression assay by analysis of GUS activity was performed to analyze promoter activation (**A**); the relative levels of *chs*_H1 mRNA were determined by RT qPCR and normalized against the elongation factor 1 housekeeping gene to analyze the activation of *chs*_H1 gene by the TFs (**B**). Infiltrated *A. tumefaciens *strains in panel A: C-control, infiltration of *A. tumefaciens *LBA 4404 without plant vector; M2, *Hl*Myb2; s-M3; s-*Hl*Myb3; +W1, combinations with *Hl*WDR1; +B2, combinations with *Hl*bHLH2; +B2W1, combinations with HlbHLH2 and *Hl*WDR1, 35S:GUS, ß-glucuronidase driven from 35S promoter of CaMV. The samples in panel B: 1, control infiltration of LBA 4404; 2, *chs*_H1 gene; 3, *chs*_H1 gene plus TF combination *Hl*Myb2/*Hl*bHLH2/*Hl*WDR1; 4, *chs*_H1 gene plus TFs in combination s-*Hl*Myb3/*Hl*bHLH2/*Hl*WDR1. Bars represent the confidence intervals at level α = 0.05.

According to our results, a weak activation of P*chs*_H1 was observed after co-infiltration with *Hl*Myb2 (Figure 3). A very weak activation signal on the level of 40 pmol MU/mg/min was found for *Hl*Myb3 in accordance to our previous study [21]. A similar very low activation of P*chs*_H1 was found for *Hl*bHLH2, despite the presence of two bHLH G-boxes (Figure 6A), and no activation exceeding the background signal was observed when *Hl*WDR1 TF was applied by itself (not shown). However, dramatic increases of the activation of P*chs*_H1 were observed both in Myb/bHLH binary and ternary combinations of the TFs under investigation (Figure 7A). The ternary combination led to 1721 ± 315 pmol MU/mg/min for M3B2W1 and 5089 ± 107 pmol MU/mg/min for M2B2W1 (Figure 7A). The M2B2W1 combination significantly exceeded the activation caused by the strong constitutive 35S promoter of CaMV (1658 ± 102 pmol MU/mg/min).

In order to assay the real activation of transcription of *chs*_H1 genes having very conserved promoter regions [16] we performed the co-infiltration of TFs with the "native" *chs*_H1 gene containing the natural intron sequence (Figure 6B) as described earlier [16]. The relative expression levels of *chs*_H1 mRNA were then measured by RT qPCR and 35S:*chs*_H1 was used as a reference (Figure 7B). In correspondence to the previous results, the ternary combinations M3B2W1 and M2B2W1 were found to strongly activate the natural *chs*_H1 gene construct thereby showing good accordance between both assays.

To enable the characterization of the binding sites responsible for the strong activation observed with both M3B2W1 and M2B2W1, a series of truncated variants of P*chs*_H1 lacking the predicted TF-binding boxes were inserted (Figure 6A) in the GUS reference vector (Figure 6B) and assayed in a transient expression system (Table 1). The majority of promoter activity (about 50%) was found to depend on the presence of the MybP box, although a significant activity (about 50%) was still observed in variant 2 containing the Myb-like box, Myb-P like box, Myb-C box and two G-boxes (Figure 6A, Table 1). By removal of the boxes, the promoter activity is gradually decreasing in a similar fashion for both ternary combinations to the low activity of construct 5 containing a single G-box. In order to confirm the high dependency of P*chs*_H1 on the Myb-P box, we changed the sequence TCCTACC by a triple mutation to TAACACC (mutated nucleotides underlined) (Table 1). A decrease of about 50% was observed for both ternary combinations. Despite the critical role of the Myb-P box, a ΔP*chs*_H1 variant containing the Myb-P box attached upstream of the TATA box (Figure 6A) was inactive, suggesting the dependence of P*chs*_H1 activation on the length and/or specific order in the promoter sequence.

**Table 1:** Activation of modified variants of P*chs*_H1 by hop TFs^§^

Promoter variant*	length (bp)	Combinations M2B2W1	M3B2W1
P1*chs*_H1	500	100.0***	100.0***

P^Mut^*chs*_H1**	500	56.3 ± 26.0	48.0 ± 8.8

P2*chs*_H1	436	52.2 ± 16.7	40.7 ± 22.1

P3*chs*_H1	346	41.7 ± 18.8	21.1 ± 2.0

P4*chs*_H1	297	12.2 ± 2.3	12.2 ± 7.0

P5*chs*_H1	240	3.5 ± 0.6	2.1 ± 0.1

P6*chs*_H1	217	0.4 ± 0.1	0.3 ± 0.1

PΔ*chs*_H1	271	0.7 ± 0.2	0.6 ± 0.1

BGF-0^#^	0	<< 0.1	<< 0.1

P35S:BGF^##^	681	29.2 ± 15.6	87.5 ± 5.3

§ Supplemental GUS histochemical analysis together with graph containing GUS activity values is presented in additional file 8.

* for details see Figure 6A; ** Myb-P box was mutated; *** 100% was 5089 ± 84 and 1721 ± 315 pmol MU/mg/min for M2B2W1 and M3B2W1, respectively

# infiltration with promoterless BGF-0 vector [58] (negative control, background); ## infiltration with p35S:BGF vector (35S control - see Methods); the average activity measured for p35S:GUS without co-infiltration with MBW TFs was 1658 ± 102 pmol MU/mg/min

### Substitution and complementation of activating complexes with selected *H. Lupulus*- and *A. Thaliana*-derived myb TFs

To investigate a possible functional complementation, related Myb TFs from *H. lupulus *and *A. thaliana *were compared using the transient expression system. In addition to P*chs*_H1, also the promoter P*chs*4 of the chalcone synthase-like gene from hop was analyzed. The gene for *chs*4 was cloned previously [GenBank: AJ430353] by Novák et al. [18]. The P*chs*4 sequence is 629 bp in length and has a different architecture in comparison to P*chs*_H1, while still containing similar Myb and bHLH boxes. Using the PLACE database it was possible to identify a MybP*Zm *box core CCWACC (sequence CCAACC) [53] at position -101 to -95, a Myb P-like box corresponding to the consensus sequence MACCWAMC (sequence AACCTAAC) at position -92 to -85 [55] and another potential Myb1AT box with the consensus sequence WAACCA [59] at position -212 to -207 (sequence TAACCA). One bHLH G-binding box (sequence CACGTG) and one E-binding box (CACATG) [60] were found at positions -132 to -127 and -120 to -115, respectively.

The relative activation levels for the individual TF combinations are shown in Table 2. It was observed that the P*chs*_H1 promoter is most strongly responding to the ternary combinations, whereas the strongest response of P*chs*4 was found for independent Myb action or binary combinations. Furthermore, *A. thaliana-*derived *At*Myb23 (*At5g40330*) and *At*Myb75 (*At*PAP1, *At1g56650*) clearly supplemented the M component in the ternary MBW combination, while *At*Myb12 did not cause any enhancement of activation in the MBW combination over the MB or MW combinations. *At*Myb12 also strongly activated P*chs*4 when applied alone or in the MW combination. *At*Myb75 and *Hl*Myb2, the strongest ternary activators of P*chs*_H1, showed low capability to activate P*chs*4. Interestingly, a clear-cut difference was observed for the subvariants of *Hl*Myb3 [20]. While *l-Hl*Myb3 shows a maximum of activation for P*chs*4 in the MB combination, the maximum of activation of P*chs*_H1 was observed for the shorter variant s-*Hl*Myb3 in the MBW combination. Both *Hl*Myb3 subvariants show a significant effect on both promoters in the MB combinations (Table 2, see also Figure 7) which is even more pronounced for P*chs*4 (Table 2). *Hl*Myb1 [19] appeared as the weakest activator of P*chs*_H1, although still an induction of 304 ± 47 pmol GUS/mg/min was observed for the MBW combination. Finally, although *Hl*Myb7 shows some similarity to the other investigated Mybs (Figure 1), it is not an activator in any combination, neither for P*chs_*H1 nor for P*chs*4.

**Table 2:** Activation of promoters P*chs*_H1 and P*chs*4 with selected Myb and hop TFs in various combinations

Myb (M)	AC	M	MB*	MW**	MB*W**
				**P*chs*_H1**	

*Hl*Myb2	FN646081	(+)	+	(+)	++

*s-Hl*Myb3	AM501509	0	+	0	++

*l-Hl*Myb3	AM501509	0	+	0	0

*Hl*Myb1^#^	AJ876882	0	(+)	n.d.	(++)

*Hl*Myb7	AB292244	0	0	0	0

*At*Myb23	NM_123397	(+)	(+)	+	++

*At*Myb12	NM_130314	++	++	++	0

*At*Myb75^#^	NM_104541	+	+	+	++

				P*chs*4	

*Hl*Myb2	FN646081	0	0	0	(+)

*s-Hl*Myb3	AM501509	+	+	+	0

*l-Hl*Myb3	AM501509	+	(++)	0	0

*Hl*Myb7	AB292244	0	0	0	0

*At*Myb23	NM_123397	0	0	(+)	0

*At*Myb12	NM_130314	+	0	+	0

*At*Myb75^$^	NM_104541	0	(+)	0	(+)

* B = *Hl*bHLH2 (AC: FR751553)

**W = *Hl*WD40_1 (AC: FN989721)

^$ ^*At*PAP1;

^#^not tested for P*chs*4

n.d. not determined

(+) up to 10% of maximal level

+ up to 70% of maximal level

++ maximum activity observed; (++) low maximal activity; the maximal levels for M/promoter/combination/pmol GUS/mg/min: *Hl*Myb2/P*chs*H1/MBW/5127; s-*Hl*Myb3/P*chs*H1/MBW/1192; l-*Hl*Myb3/P*chs*4/MB/511; *Hl*Myb1/P*chs*_H1/MBW/304; A*t*Myb23/P_*chs*H1/MBW/2036; *At*Myb12/P_*chs*H1/MW/3108; *At*Myb75/P*chs*_H1/MBW/5786

0 = no increase of GUS activity over the background caused by M; no enhancement caused by MB or MW over M; no enhancement caused by MBW over M or over the binary combinations.

### HlMyb7 is a suppressor of promoter activation

**Figure 8:**
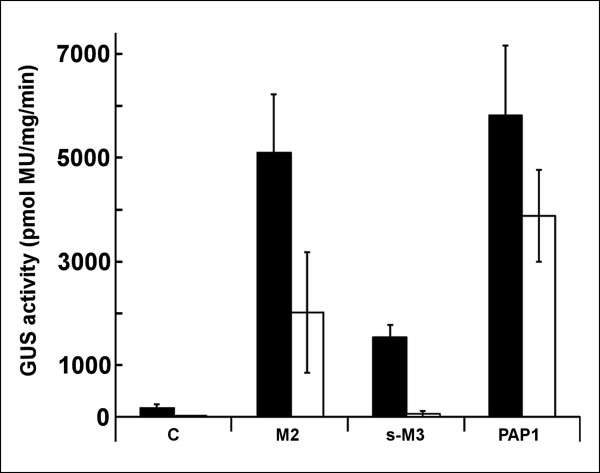
**Suppressor action of *Hl*Myb7 on P*chs*_H1 activation with TFs in various combinations**. The combinatorial transient expression assay was performed and equal agrobacterium aliquots were mixed. For suppressor activity an aliquot of *A. tumefaciens *bearing 35S::*HlMyb7 *was added to the following combinations: M2, *HlMyb2*/*HlbHLH2*/*HlWDR1*; s-M3, s-*HlMyb3*/*HlbHLH2*/*HlWDR1*; PAP1, *AtPAP1*/*HlbHLH2*/*HlWDR1*(white columns). Black columns, activity levels without *HlMyb7*. Instead of suppressor, LBA 4404 was added. C - control, infiltration with *A. tumefaciens *LBA 4404 without plant vector. Bars represent the confidence intervals at level α = 0.05.

Although *Hl*Myb7 is related to the other investigated Mybs, especially to *At*Myb12 and *Hl*Myb2, this hop-derived transcriptional regulator showed no ability to activate the hop *chs *promoters (Figure 1). As we identified a transcriptional suppressor motif PDLNLELRIS, in the C terminal region of *Hl*Myb7, the suppressive activity of *Hl*Myb7 was investigated by assaying P*chs*_H1 activity in transient expression systems containing MBW × *Hl*Myb7 combinations with *Hl*Myb2, s-*Hl*Myb3 and *At*Myb75 (PAP1) (Figure 8). The addition of *Hl*Myb7 was found to significantly decrease the reference GUS activity. A similar suppression by *Hl*Myb7 in the range of 50 to 90% was observed for binary combinations, as well as for the independent action of individual Myb genes on both *chs *promoters (data not shown), suggesting a more wide suppressive activity of this Myb. In order to analyze the possible role of the Myb-P boxes in this inhibition, the P2*chs*_H1 construct (Table 1) was used to analyze the influence of *Hl*Myb7 to the promoter activity after co-infiltration with M2B2W1 × *Hl*Myb7, s-M3B2W1 × *Hl*Myb7 and corresponding controls without *Hl*Myb7. As the suppressive activity was also found to reach about 50% (data not shown), it is most likely that the Myb-P box does not play a major role.

### Influence of cloned TFs on the accumulation of anthocyanins in the heterologous system of *petunia hybrida *leaves

The hop-derived TFs investigated in this study are all related to known transcription factors involved in regulation of flavonoid pathways. Because of the significant activation of *chs*_H1 genes, the hop-derived TFs are expected to increase the pool of naringenin chalcone, whereby they may stimulate downstream anthocyanin biosynthesis and accumulation. In the lupulin glands, however, colored anthocyanins are not detectable as it is most likely that chalcone isomerase and further downstream enzymes of anthocyanin biosynthesis are not expressed. Instead, lupulin glands accumulate prenylated chalcones and other terpenophenolics composing the highly specific lupulin metabolome [52]. On the other hand, accumulation of naringenin chalcone in *P. hybrida *may lead to accumulation of anthocyanins. To further investigate the specificity of the cloned hop TFs in different combinations, as well as their potential to activate anthocyanin pathway, a transient TF expression system in *P. hybrida *was used. For comparison, *At*PAP1 was included as a regulator having the capability to co-activate the anthocyanin biosynthesis in petunia leaves providing co-expression of *AtPAP1 *and *chs*_H1 genes arranged in tandem [16]). Hence, *At*PAP1 or/and hop *chs*_H1 activators, e.g., s-*Hl*Myb3, *Hl*Myb2, *Hl*bHLH2 and *Hl*WDR1 (Figure 7) in particular combinations were analyzed for possible anthocyanin induction.

**Figure 9:**
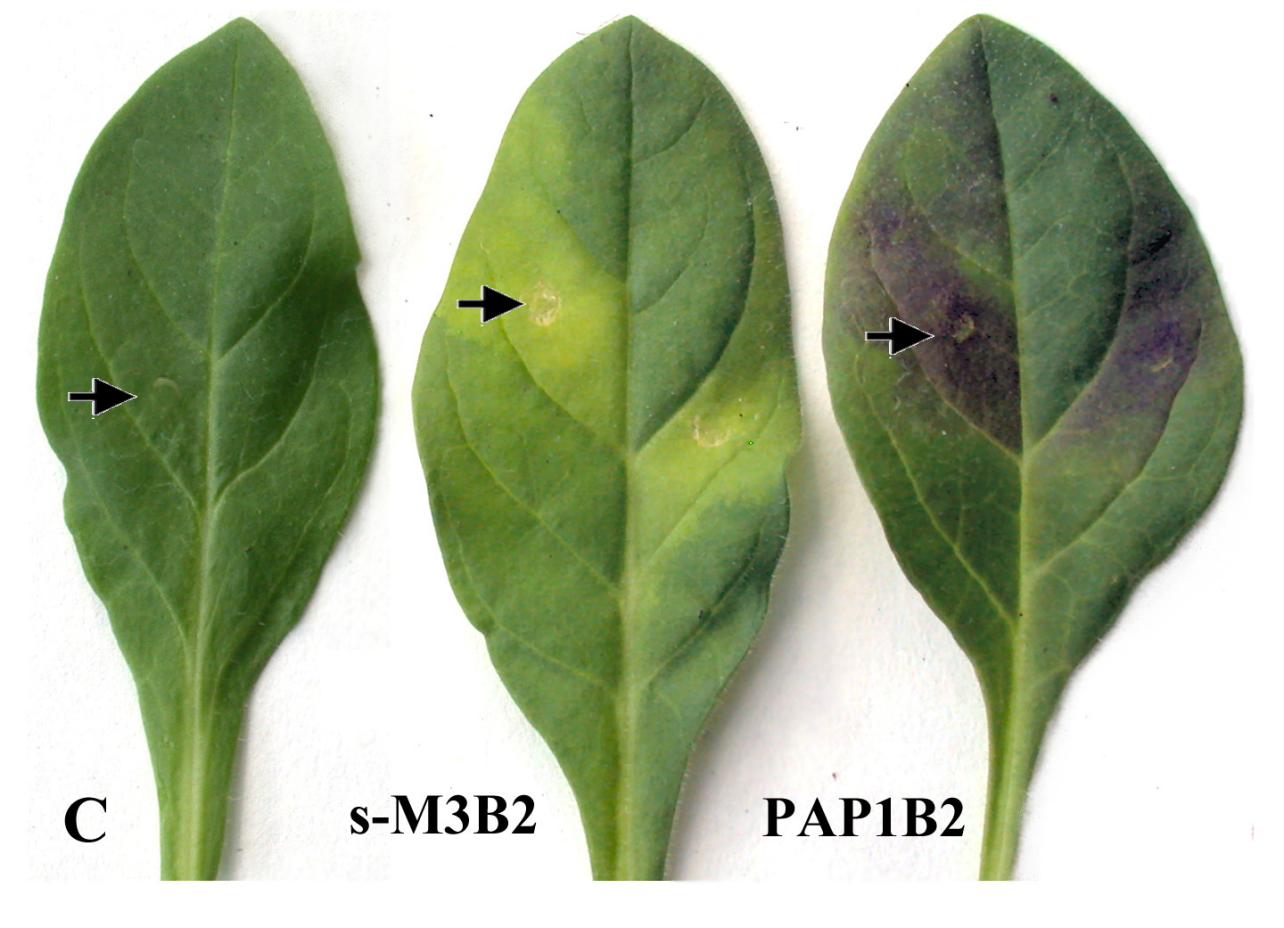
**An example of anthocyanin pathway activation in *Petunia hybrida *leaves after infiltration with different TF combinations**. The vector pLV-67 (see: Figure 6B, III) was infiltrated either in combination with s-*HlMyb3 *and *HlbHLH2 *(s-M3B2) or *AtPAP1 *and *HlbHLH2 *(PAP1B2), mixed in equal amounts. The yellowish spots in the s-M3B2 variant and the anthocyanin spots in the PAP1B2 leaves developed ca. three days post-infiltration. The sites of infiltration are indicated by arrows.

It was shown that only the co-infiltration of the *chs*_H1 gene (Figure 6B, construct III) with *AtPAP1 *and *HlbHLH2 *was found to strongly induce an accumulation of blue anthocyanins in the petunia leaves (Figure 9, for HPLC results see Additional file 5). This may be due to both an increased pool of chalcones and the activation of downstream anthocyanin biosynthesis genes. Infiltration of either *AtPAP1 *or *HlbHLH2 *alone did not result in any detectable level of anthocyanins (Additional file 5). Based on retention times and UV-VIS spectra, the profile of the induced leaf anthocyanin metabolites was found to be qualitatively very similar to the profile of anthocyanins found in the blue corolla tissue of petunia flowers. However, significant differences were found in quantitative composition. It is surprising that only minor amounts of anthocyanin metabolites were observed for the ternary combination *At*PAP1B2W1 that led to dramatic activation of P*chs*_H1 as shown above (Table 2). Neither the combinations with hop-derived Mybs, *Hl*Myb2 or *s-Hl*Myb3 that activated P*chs*_H1, i.e., *s-*M3B2, *s-*M3B2W1 nor M2B2W1 resulted in blue pigmentation in the petunia leaves. The metabolically highly active *s-Hl*Myb3 [20], caused yellow spots on the leaves in combination with *Hl*bHLH2 (Figure 9). The hop TFs, *Hl*Myb2, *s-Hl*Myb3 and WDR1 were not able to co-induce anthocyanin biosynthesis in petunia leaves.

## Discussion

### Sequences and functional properties of lupulin gland-associated transcription factors and their potential role as regulators of the biosynthesis of the lupulin metabolome

Four new transcriptional regulators, i.e., *Hl*Myb2, *Hl*Myb7, *Hl*bHLH2, and *Hl*WDR1, were isolated and cloned from the Czech hop variety, Osvald's 72 in this study. All these TFs show a highly specific expression in the lupulin glands and display high similarity to established Myb, bHLH and WDR TFs, that are known regulators of the flavonoid biosynthetic pathway in various plant species. Some of these regulators of flavonoid biosynthesis [24,61] form ternary MBW activation complexes like (PhAN2/AN1/AN11) in *P. hybrida *[29] and (AtTT2/TT8/TTG1) in *A. thaliana *[28] as well as recently described TF complexes in peas [30] and *Lotus japonicus *[31]. MBW complexes have been described to regulate not only pigmentation, but also the fate of epidermal cells, including the initiation and further development of trichomes [26,62]. Hop lupulin glands are glandular trichomes and, therefore, it was anticipated from the evolutionary point of view, that the combinatorial MBW machinery could be involved in the activation of genes related to the lupulin metabolome biosynthesis. Using a combinatorial transient expression system, it was shown that a M2B2W1 combination synergistically induced a very strong activation of native *chs*_H1. This gene is coding for a crucial hop "true" chalcone synthase endowed with the ability to produce naringenin chalcone with a high catalytical rate [16] and, therefore, it is likely playing an important role in the biosynthetic pathway leading to the accumulation of prenylated chalcones in lupulin glands, including xanthohumol and desmethylxanthohumol (a direct precursor of the potent phytoestrogen 8-PN) [14,16].

The newly isolated *Hl*Myb2 shows a typical sequence motif [DE]Lx2[RK]x3Lx6Lx3R within the R3 domain that is characteristic for a possible interaction with a member of the sub-group of IIIf bHLHs [24,38,46]. This finding is in accordance with the ability of *Hl*Myb2 to strongly activate P*chs*_H1 in a M2B2W1 combination using a combinatorial transient expression system. On the other hand, clear synergistic effects were also observed for subvariants of the polyfunctional *Hl*Myb3 (cloned and characterized previously [20]), although the bHLH-interacting motif is missing in its amino acid sequence. Especially the shorter subvariant s-*Hl*Myb3 resulted in a significant activation of the *chs*_H1 gene in both binary s-M3B2 and ternary s-M3B2W1 combinations. This suggests that *Hl*bHLH2 is able to interact with s-*Hl*Myb3 by either involving alternative hydrophobic residues in these positions exposed on the protein surface, or some alternative way. According to our unpublished results, Electrophoretic mobility shift assay (EMSA) showed some physical interaction between radioactively labeled P*chs*_H1 and proteins extracted from leaves infiltrated with s-M3B2 and s-M3B2W1 complexes. More experiments are necessary to clarify this interaction. The different length of the N-terminal domain of *Hl*Myb3 has a crucial effect on its ability to activate P*chs*_H1 in M3B2 and M3B2W1 combinations, as practically no synergism was observed for the l-M3B2W1 combination. The divergent effects of overexpressed l- and s-*Hl*Myb3 TFs was observed earlier as reflected by changes in the composition of metabolites in petunia leaves, as well as in changes of the morphogenesis of *A. thaliana *and *P. hybrida *transgenotes [20]. The functional analyses presented in Table 2 confirmed the complementation of hop lupulin gland-specific Mybs by selected *A. thaliana *Mybs in various binary or ternary combinations, suggesting not only sequential but also functional similarity of these TFs. For instance, *At*PAP1 in combination with *Hl*bHLH2 and *Hl*WDR1 resulted in a strong synergistic effect. Interplay between PAP1 and other components of MBW complexes in *A. thaliana *has been described previously [28]. In our experiments the highest synergism in the ternary MBW combination was also observed with *At*Myb23, while the flavonoid regulator *At*Myb12 responded synergistically only to the binary BM and BW combinations, suggesting some specificity of protein:protein interactions among these TFs. In addition to R2R3 Mybs, we confirmed the functional complementation of *Hl*WDR1 by TTG1 from *A. thaliana *(not shown).

The newly cloned *Hl*Myb7 appears to be a potential negative R2R3Myb regulator of both *chs*_H1 and *chs*4 genes in hop as it showed significant suppressor activity in a transient expression system. This is consistent with the identification of a PDLNLELRIS sequence in the C-terminal part of this protein conforming to the consensus pdLNLD/ELxiG/S sequence characteristic for subgroup 4 R2R3Mybs [63], which is conserved in repression region of the *At*Myb4 repressor [42]. The repression caused by *Hl*Myb7 was found to be independent on the Myb-P box positioned on the 5'region of P*chs*_H1, but it remains to be established whether it exerts its repressive activity via an interaction with MBW components, other proteins or binding to other elements in the promoter sequence.

Upregulation of *chs*_H1 genes by both s-M3B2W1 and M2B2W1 combinations may increase the pool of available naringenin chalcone-precursors for further production of lupulin prenylchalcones co-determined by expression of downstream hop genes including prenyltransferase [64], and *O*-methyl transferase 1 [41]. While hop transformed by a single *AtPAP1 *gene was characterized by a significantly higher production of anthocyanins, rutin and isoquercetrin in cones and flowers in comparison to wild type plants [65], anthocyanins were not accumulated in the lupulin glands [52], most likely due to the lack of expression of the specific enzymatic machinery in these specialized structures. Conversely, hop TFs in s-M3B2W1 and M2B2W1 combinations were unable to co-activate the anthocyanin pathway in *P. hybrida *as found for the *At*PAP1B2 combination, thereby supporting the idea about functional specialization of lupulin-specific TFs. According to our unpublished results, neither the s-M3B2W1 nor the M2B2W1 combination was able to induce other promoters of hop genes like valerophenone synthase or the *O*-methyl transferase1 (P*omt*1) [21], confirming its specificity for the *chs*_H1 gene.

Neither the ternary combinations s-M3B2W1, M2B2W1 nor the P*chs*_H1-activating s-M3B2, M2B2 complexes were able to induce anthocyanin pigments in petunia leaves. The significantly elevated level of leaf metabolites and blue anthocyanins resulting from the *At*PAP1B2 combination is probably due to an ability of *At*PAP1B2 complex to activate also downstream genes in petunia, like chalcone isomerase (CHI), flavanone 3-hydroxylase (F3H), dihydroflavonol 4-reductase (DFR), and anthocyanidin synthase (ANS) [66]. Indeed, the metabolome and mRNA screenings shown that the overexpression of the *At*PAP1 gene is resulting in the activation of at least 25 petunia genes and also some new metabolites were produced [67]. Thus, further investigations are required to clarify the profile of hop genes that may be up-regulated by combinatorial action of isolated hop TFs.

### Proposed regulation of hop *chs *genes as predicted from combinatorial analysis

**Figure 10:**
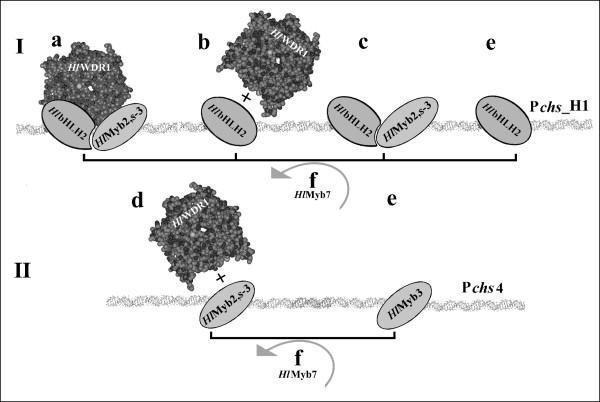
**Simplified scheme of P*chs*_H1 (I) and P*chs*4 (II) activation with combinations of hop TFs as observed in heterologous system of *N. benthamiana***. The basic scheme was accommodated from Baudry et al. [27]. 3-D model of *Hl*WDR1 in the scheme was obtained using SWISS-MODEL Workspace (see Methods). (**a**) the whole ternary complexes consisting of MYB, bHLH and WDR TFs; (**b**) bHLH and WDR binary combination; (**c**) MYB and bHLH combinations; (**d**) Myb and WDR combinations; (**e**) "direct" independent action of hop TFs that does not require complex with analyzed hop TFs; (**f**) inhibitory effect of HlMyb7. The figure is not in scale, TFs are assumed to interact with promoter binding sites. Binding boxes are not shown in the scheme. The weak signals close to background interactions are not considered in the scheme.

To analyze the functional properties of the newly cloned hop TFs it was opted to use a combinatorial transient expression system because hop transformation [68] and cultivation to reach flowering are very laborious and time-consuming especially for the investigation of the TFs in various combinations. While this transient expression assay is characterized by a negligible background signal i.e. signal originating from endogenous *N. benthamiana *TFs [21], it enabled us to efficiently compare the activation of different promoter constructs by various combinations of hop TFs. A comparison of truncated promoter constructs revealed a major dependence of P*chs*_H1 activation by s-M3B2W1 and M2B2W1 combinations on a single Myb-P box characteristic for binding the P1 TF of *Zea mays *[53]. However, other binding boxes such as the Myb-like box, the Myb-P-related box, the MYB IIG-like box and the G-binding sites characteristic for bHLH G-box binders driving the flavonoid pathway [23] also contribute to the overall activity. Therefore, each component of the promoter seems to impact on the degree of activation and it is the integral composition of the promoter that dictates the involvement of particular TFs in specific combinations. This is apparent on a comparison of two *chs *promoters both driving chalcone synthases having high expression in lupulin glands, i.e., *chs*_H1 and *chs*4 (see Figure 7, Table 2). A proposed schematic overview of the interactions of the investigated TFs with the different promoters is shown in Figure 10. The two promoters having a different spatial composition of similar potential Myb and bHLH binding boxes are activated differently by the same transcription factors. P*chs*_H1 is predominantly activated not only by M2B2W1/s-M3B2W1 ternary complexes, but also by B2W1 and MB binary combinations and by action of *Hl*bHLH2 independent on hop B2 and W1 (Figure 10a,b,c,e). Single Myb and MW binary combinations did not result in activation of P*chs*_H1. This is in sharp contrast with P*chs*4, where "direct activation" by *Hl*Myb3 or the binary s-M3W1 combination is preferred (Figure 10d,e). Activation of P*chs*4 by the M2B2W1 ternary complex is negligible (see Table 2), as well as any combination including *Hl*bHLH2 (Figure 10). Interestingly, both promoters can be negatively regulated by the *Hl*Myb7 repressor (Figure 8; Figure 10). The general dependence of the combinatorial action of TFs on the specific promoter composition is illustrated in many studies summarized in recent reviews [23,24,69]. Although we did not observe any significant activation of P*chs*_H1 with *N. benthamiana *TFs, we cannot exclude some influence of endogenous *N. benthamiana *TFs on the combinatorial regulation of P*chs*_H1 in this heterologous system. Further study will be conducted to confirm proposed regulation pattern. The specific co-regulation of the lupulin metabolome biosynthesis genes depends on the steady state levels of individual TFs forming the actual TF balance. It is, therefore, conceivable that misbalancing of such complexes like M2B2W1 by the downregulation of a specific TF complex component could lead to a significant decrease in gene expression and thus in the production of the lupulin metabolome. In support of this idea, it should be noticed that we recently detected a misbalanced expression of TFs in decolored petioles of diseased hop plants infected with hop stunt viroid (HSVd) (unpublished).

## Conclusions

In the present study new TF homologues corresponding to the plant R2R3Myb, bHLH and WDR families were cloned using a cDNA library derived from hop (*Humulus lupulus *L.) lupulin gland tissue. The cloned *Hl*Myb2 and -7, *Hl*bHLH2 and *Hl*WDR1 TFs display a high similarity to known TFs regulating the flavonoid biosynthesis pathway and were found to be highly specifically expressed in lupulin glands. The functional activity of these TFs was investigated using a combinatorial transient expression system in infiltrated leaf sectors of *Nicotiana benthamiana*. These experiments provided new insight into the complexity of the regulation of *chs_*H1 genes, as well as into the differential activation of *chs*_H1 promoter and the lupulin-specific chalcone synthase 4 promoter. The regulation involves variations of ternary, binary and independent TFs action. Complementation of hop and *A. thaliana *TFs was shown in particular combinations. The hop chs-activating TFs, *Hl*Myb2 and 3, *Hl*bHLH2 and *Hl*WDR1 in various combinations did not enable the induction of the anthocyanin pathway in *P. hybrida *suggesting the specialization of the MBW machinery in the specialized tissue of hop glandular trichomes. *Hl*Myb7 was characterized as an R2R3 repressor that could act as a potential co-regulator of the lupulin metabolome biosynthesis.

## Methods

### Plant material and plant cultivation conditions

The Czech semi-early red-bine hop Osvald's clone 72 was grown under natural field conditions in the world hop collection of the Hop Research Institute in Žatec (Northern Bohemia). *Petunia hybrida *cv. Andrea and *Nicotiana benthamiana *plants were maintained in the green house at a temperature of 25 ± 3°C. Plants were grown under natural light conditions with supplementary illumination [170 μmol m-2 s-1 PAR] to maintain a 16 h day period.

### Isolation and purification of lupulin glands, preparation of cDNA library and cloning of transcription factor cDNAs

Lupulin glands were isolated from fully matured cones of the hop variety Osvald's clone 72 collected in the first half of August 2009 and 2010, two weeks before harvest using the procedure according to Nagel et al. [41]. Isolated lupulin glands were used for RNA extraction, purification and hop cDNA library construction according to manufacturers' instructions. Briefly, glandular tissue was frozen in liquid nitrogen and grinded in the Concert Plant RNA Reagent (Invitrogen). Then cell debris was removed by centrifugation and chloroform treatment, followed by RNA precipitation using isopropanol. Dissolved RNA purification on columns with DNaseI treatment was carried out according to the RNeasy Plant Mini Kit procedure (Qiagen), and subsequently mRNA was isolated with the Dynabeads mRNA Purification Kit (Invitrogen Dynal). Purified mRNA samples were used for cDNA library construction and lambda packaging, according to the ZAP-cDNA Synthesis Kit and the ZAP-cDNA Gigapack III Gold Packaging Kit instructions (Stratagene), respectively.

The motifs for cloning of TFs cDNA were obtained by screening the *Humulus lupulus *sequences in both the GenBank EST (http://www.ncbi.nlm.nih.gov) and TrichOME (http://trichome.noble.org/trichomedb/) databases. In total, 258,472 ESTs were screened for R2R3Myb, bHLH and WDR analogues using the Advanced BLAST 2.0 script (http://blast.ncbi.nlm.nih.gov/). The obtained EST sequences were used for the design of primer sequences to be used for screening of the lupulin gland-specific cDNA library. High fidelity Pwo polymerase (Roche Molecular Biochemicals) was used for amplification.

*Hl*Myb2 (clone 2962) was amplified from the library using primer combinations "Myb2 start" and "Myb2 stop", *Hl*Myb7 (clone 2265) was amplified using primers "Myb7 start" and "Myb7 stop" and for *Hl*bHLH2 (clone 3497) amplification we used the primers "bHLH start" and "bHLH stop". All primer sequences are listed in Additional file 6. For amplification of *Hl*WDR1(3140), a two-step amplification reaction was used. First, a mixture of cDNA fragments was obtained from the library using the primer combination "WDR start" and 3' oligo d(T)-anchor primer (5'/3' RACE Kit 2^nd ^generation kit from Roche Diagnostics) and then a nested reaction was performed using a "WDRnes" primer together with "PCR WDR anchor" primer. A fragment having the length of approximately 1.6 kb was then cloned.

cDNAs of *At*Myb12 and *At*Myb23 were amplified from RNA of *A. thaliana *var. Columbia using the Titan One Tube RT-PCR (Molecular Biochemicals) including a high fidelity *Pwo *polymerase (Roche Molecular Biochemicals) and primers derived from sequences described in the GenBank database (see Additional file 6).

### Genomic DNA preparation and genomic blots

Hop genomic DNA was prepared by the CTAB method [70]. 5 μg DNA from each plant was digested with 50 units of *Eco*RI, *Bam*HI, *Xba*I, *Xho*I, *Bgl*II, or *Pst*I in 150 μl reaction mixes that were incubated overnight at 37°C. Digested DNA was subjected to electrophoresis and blotted as described previously [21] Pre-hybridization (2 h at 65°C in 30 ml) and hybridization (overnight at 65°C in 20 ml) were carried out in buffer according to Church and Gilbert [71] using a hybridization oven. The membranes were hybridized to *HlWDR1, HlbHLH2 *or *HlMyb2 *full length cDNA probes having a specific activity 5 × 10^7 ^cpm/μg DNA. Hybridization signals were detected by phosphorimaging using a Typhoon 9200 imager (Amersham Pharmacia).

### RNA isolation and real-time PCR analyses

For real-time quantitative PCR (RT qPCR) and for the reverse transcriptase-polymerase chain reaction (RT-PCR), total RNA was isolated from 100 mg of plant leaf tissue using Concert™Plant RNA Reagent (Invitrogen) following RNA purification and DNA cleavage on columns (RNeasy Plant Total RNA kit, Qiagen). The primer combinations for the RT qPCR of the hop TFs are listed in Additional file 6. All these primers had an annealing temperature close to 58°C and amplified unique, non-conserved regions of the TFs. Four micrograms of total RNA were reverse transcribed using oligo dT18 primer and SuperscriptII reverse transcriptase (Invitrogen) at 42°C for 60 min. A total of 5 μl of 50 × diluted cDNA was used for a 20 μl PCR reaction with 0.6 units of Hot Start Ex Taq polymerase (TaKaRa Bio), Taq buffer 1 ×, dNTPs 200 μM each, SYBR Green 1:20,000 (Molecular Probes) and primers 375 nM each. All amplifications were carried out on a Bio-Rad IQ5 cycler for 40 cycles (94°C for 20 s, 59°C for 30 s, 72°C for 30 s) following an initial denaturation/Taq activation step (94°C for 5 min). The product size was confirmed by melting analysis and 2% agarose gel electrophoresis. Data were analyzed and quantified with the Bio-Rad IQ5 Optical System version 2.0 software. The abundance of a reference transcript, glyceraldehyde-3-phosphate dehydrogenase (GAPDH) [41,50,51], was estimated in parallel in each sample. GAPDH was amplified using the primer combination "HL-GAP-F1" and "HL-GAP-R1" as reported previously [21]. The relative values were standardized to GAPDH with the "Delta-delta method"and normalized to the sample with highest expression (calibrator, set to 100%) according to Pfaffl [72]. The data points show the means ± S.D. of two replicates of each PCR reaction. For RT-PCR amplifications we used cDNA primers as above and the high-fidelity Titan One Tube RT-PCR kit (Roche Molecular Biochemicals).

### Construction of plant expression vectors and preparation of *chs*_H1 promoter subvariants

TFs *HlMyb2, HlMyb7, HlbHLH2 *and *HlWDR1 *CDS were re-amplified with the high fidelity Pwo polymerase using the primer pair combinations (for the sequences see Additional file 6): "5'Myb2Apa" and "3'Myb2Kpn"; "5'MYB7Xho" and "5'MYB7Xba; "bHLHKpn" and "bHLHBam"; "5'HlWDXho" and "3'HlWDXba".

For the cloning of the *A. thaliana *TFs *AtMyb12 *and *AtMyb23 *we used the following combination of cloning primers: "3'Myb12Xba" and "5'Myb12Xho"; "5'Myb23Xho" and "3'Myb23Xba".

Prepared CDS fragments of *HlWDR1, HlMyb7, AtMyb12 *and *AtMyb23 *were treated with *Xho*I and *Xba*I; *HlMyb2 *with *Apa*I and *Kpn*I and *HlbHLH2 *with *Kpn*I and *Bam*H1. Gel-purified fragments were then fused to the 35S CaMV promoter by cloning into the corresponding unique sites of the intermediary vector pLV68 [16]. The whole TF cassettes containing the 35S CaMV promoter were excised using restrictases *Asc*I and *Pac*I and cloned in the plant vector pLV07 as described by Vrba and Matoušek [73]. These vectors were introduced in the *A. tumefaciens *strain LBA 4404 by the freeze-thaw method [74] and used for leaf infiltration of *N. benthamiana *or *P. hybrida *leaves as described previously [16]. The *Hl*Myb3-bearing plant vectors and *A. tumefaciens *strains were described previously [20].

The *chs*_H1 promoter sequence was isolated earlier [14] and cloned to reference GUS vector previously [16]. The 5'truncated variants (Table 2) were amplified using the 5' primers listed in Additional file 6: variant/primer: 1/1PchsH1Eco; 2/2PchsH1Ec; 3/3PchsH1Eco; 3/4PchsH1Eco; 4/4PchsH1Eco; 5/5PchsH1Eco; 6/6PchsH1Eco combined with 3' primer "Pchs1endXba". To prepare a mutated variant of the Myb-P box, the primer "mutPmyb5" was combined with the "Pchs1endXba" primer. The delta variant of P*chs*_H1 was prepared using a combination of "deltaPmyb5" and the "Pchs1endXba" primer. In this case variant 6 of the promoter served as a PCR template. The promoter P*chs*4 (600 bp) was amplified from the *chs*4 gene [GenBank:AJ430353] [18] (clone 1746) using primers "Pchs4Eco" and "Pchs4Xba". The promoter regions were then fused to GFP/GUS genes by cloning in the unique *Eco*RI and *Xba*I restriction sites of the plant vector pBGF-0 [58] as described earlier [16]. The infiltration with the vector pBGF-0 was used as negative control, while p35SBGF construct (clone 3771) containing 35S:GUS/GFP fusion cassette was used as p35S control. Summary of the strains of *A. tumefaciens *used and the prepared T-DNA constructs can be found in the Additional file 7.

### Combinatorial transient expression system and GUS assays

For co-infiltration, agrobacteria suspensions were prepared at an OD_600 _= 1.0 and equal volumes of each suspension were mixed prior to infiltration. *N. benthamiana *seedlings were sown in flats and grown under greenhouse conditions (22°C - 24°C) for 18 to 21 days. *A. tumefaciens *cells carrying appropriate gene constructs were activated and injected into leaf blades as described previously [21]. The activity of ß-glucuronidase (GUS) was assayed in homogenized tissues of treated leaf sectors 3-4 d following the infiltration using a fluorometric assay [75] modified by Matoušek et al. [21]. The amount of the released fluorescent dye 4-methylumbelliferone (MU) was measured using the VersaFluor™ fluorometer (Bio-Rad) with excitation at 365 nm and emission measured at 455 nm. The fluorometer was calibrated with a fresh preparation of MU (100 nM) as standard. GUS activity was expressed in pmol MU .mg^-1 ^fresh tissue .min^-1^. At least three independent repeated experiments were performed in each experimental variant. The data were statistically analyzed using Microsoft Excel 2010 software.

### Analysis of anthocyanin metabolites

Leaves of *P. hybrida *were lyophilized prior to analysis of anthocyanins. After grounding up lyophilized plant material in a mortar, samples of 10-20 mg were extracted in eppendorfs using 1 mL of methanol/acetic acid/water (9/1/10, v/v/v) and, after sonication, the extraction mixtures were kept for 16 h at 4°C. After centrifugation at 18000 rpm for 10 min, the supernatant was analyzed by high performance liquid chromatography (HPLC) using a Waters Alliance 2695 Separation Module (Waters, Zellik, Belgium) equipped with a 996 photodiode array detector (PDA). The column was an Agilent Zorbax Bonus RP (Agilent, Diegem, Belgium, 3.5 μm, 150 × 3.0 mm), while the injection volume was 10 μL. A linear gradient elution at a flow rate of 0.7 mL/min was applied using increasing ratios of solvent B (acetonitrile/acetic acid/phosphoric acid/water, 25/20/1.5/53.5 v/v/v/v) in solvent A (water/phosphoric acid, 98.5/1.5, v/v) according to the following schedule: 0 min: 25% B in A, 12 min: 35% B in A, 18 min: 48% B in A, 37 min: 100% B in A. After 5 min of washing at 100% of solvent B, the column was re-equilibrated at 25% B in A for 10 min. Chromatograms at 530 nm were extracted from the 3D-data. Peak integrations were carried out using standard parameters. Other secondary metabolites in the leaves of *P. hybrida *were analyzed according as previously described [21].

### Bioinformatic analyses

For sequence comparisons and cluster analyses we used, in addition to cloned TFs *Hl*Myb2, *Hl*Myb7, *Hl*bHLH2 and *Hl*WDR1, Myb, bHLH and WDR amino acid sequences from nucleotide and protein databases as provided by NCBI services (see Additional file 2, Additional file 3 and Additional file 4). Sequence analyses were carried out with Phylogeny Pipeline (http://phylogeny.lirmm.fr/phylo_cgi/simple_phylogeny.cgi) [76]. The trees were generated using the PhyML v. 3.0 (http://www.atgc-montpellier.fr/phyml) [77]. The generated data were visualised by FigTree v.1.3.1. (http://tree.bio.ed.ac.uk/software/figtree). Some sequence comparisons were carried out with DNASIS for Windows, version 2.6 (Hitachi Software Engineering Company, Tokyo, Japan). Amino acid classes described by [78] were used for protein comparisons. Plant *Cis*-acting Regulatory DNA elements (PLACE) database [79] and PromoterInspector option of the Genomatix (Eldorado Gene2Promoter) software (http://www.genomatix.de) were used to screen for TFs binding sites. A comparative protein analysis of the 3-D structures of the NR2R3 regions of *Hl*Myb2 and *Hl*Myb3 was performed by means of the SWISS-MODEL Workspace (http://swissmodel.expasy.org//SWISS-MODEL.htm) [80] using the loading template 1h88C.pdb. Alignments of 3-D structures and structural analyses were performed using Swiss-PdbViewer v3.7b2 [81].

## Abbreviations

8-PN: 8-prenylnaringenin; ANS: anthocyanidin synthase; B2: *Hl*bHLH2*; *DFR: dihydroflavonol 4-reductase; EMSA: electrophoretic mobility shift assay; F3H: flavanone 3-hydroxylase; GADPH: glyceraldehyde-3-phosphate dehydrogenase; GUS: ß-glucuronidase; HPLC: high performance liquid chromatography; HSVd: hop stunt viroid; CHI: chalcone isomerase; CHS: chalcone synthase; M2: *Hl*Myb2*; *M3: *Hl*Myb3; OMT: *O*-methyl transferase1; P: promoter; PLACE: Plant *Cis*-acting Regulatory DNA elements database; RT qPCR: real-time quantitative PCR; TF: transcription factor; VPS: valerophenone synthase; W1: *Hl*WDR1*; *X: Xanthohumol.

## Authors' contributions

JM as the leader of the group conceived and coordinated the project, isolated the hop TF genes from the cDNA library, cloned them into *Agrobacterium *vectors and drafted the manuscript. TK was responsible for the transient expression assays, ZF prepared the hop cDNA library, JPa screened ESTs, JPr performed real-time qPCR analyses and AH did the HPLC analysis of anthocyanins and other secondary metabolites. All authors read and approved the final manuscript.

## Supplementary Material

Additional file 1 **List of R2R3Myb TFs included in the phylogenetic tree presented in Figure 1 and alignment of amino acid sequences within R2R3 domain**. Click here for file

Additional file 2 **Comparative analysis of the 3-D protein structures of the N-terminal/R2/R3 regions of *Hl*Myb2, l-*Hl*Myb3 and s-*Hl*Myb3**. The theoretical structures were calculated and portrayed against the template 1h88C.pdb using the SWISS-MODEL Workspace. The alignments of the 3-D structures were performed using the Swiss-PdbViewer v3.7b2 (see Methods). Structures are presented as single ribbons, positions of R2 and R3 repeats are shown on alignment s-*Hl*Myb3/l-*Hl*Myb3; the positions of essential residues forming the bHLH-binding site of *Hl*Myb2 are shown on the structure alignment for s-*Hl*Myb3/*Hl*Myb2. The positions of hydrophobic amino acids substituting the bHLH-binding residues in s-*Hl*Myb3 are between brackets. The positions of the largest structural deviations are indicated on the structures by filled and hollow arrows. N and C are N- and C- terminus, respectively. Click here for file

Additional file 3 **List of bHLH TFs included in the phylogenetic tree presented in Figure 2A and alignment of amino acid sequences within bHLH domain**. Click here for file

Additional file 4 **List of WDR TFs included in the phylogenetic tree presented in Figure **3A**and alignment of amino acid sequences**. Click here for file

Additional file 5 **Relative levels of anthocyanin pigments in petunia leaves (%) infiltrated with different hop TFs or *At*PAP1 compared to anthocyanins in petunia dark blue corollas performed by HPLC analysis**. Click here for file

Additional file 6 **Oligonucleotide primers used in this study**. Table of oligonucleotide primers used in this study, arranged according the purpose for which they were designated. Click here for file

Additional file 7 **List of *Agrobacterium tumefaciens *strains and vectors**. Table of *A. tumefaciens *strains and vectors used in the analysis of lupulin gland transcription factors from R2R3Myb, bHLH and WDR families. Click here for file

Additional file 8 **Histochemical and quantitative analysis of GUS activity driven by modified variants of Pchs_H1 by hop TFs (supplement to Table **1**)**. Click here for file
