# Supplementary material for: Combinatorial analysis of lupulin gland transcription factors from R2R3Myb, bHLH and WDR families indicates a complex regulation of chs_H1 genes essential for prenylflavonoid biosynthesis in hop (Humulus Lupulus L.)
Source: BMC Plant Biol. 2012 Feb 20;12:27. doi: 10.1186/1471-2229-12-27 (PMC3340318; doi:10.1186/1471-2229-12-27)

A - List of R2R3Mybs included in the phylogenetic tree presented in Figure 1

TF name	Swiss-Prot Accession No.	Description	Proposed Biological Function	Reference*
HlMyb7	A2Q124	myb transcription factor 7 *Humulus lupulus*	flavonoid biosynthesis	-
ZmP1	P27898	Zea mays P1	anthocyanin biosynthesis	Grotewold et al. 1991
AtMyb12	O22264	transcription factor MYB12	flavonoid biosynthesis	Mehrtens et al. 2005
SmHP	D8RNQ0	Hypothetical protein Selaginella moellendorffii	unknown function	Banks et al. 2011
SbPUP	C5XXT4	Putative uncharacterized protein Sorghum bicolor Sb04g026480	unknown function	-
HlMYB1	Q4A3I7	myb transcription factor 1 Humulus lupulus	flavonoid biosynthesis	Matoušek et al. 2005
RcMyb	B9RYQ9	R2R3-myb transcription factor, putative, Ricinus communis	unknown function	-
PtMyb153	B9GXU7	Predicted protein Populus trichocarpa	unknown function	-
PtMyb009	B9GL11	Predicted protein Populus trichocarpa	unknown function	-
PtMyb123	B9GXU6	Predicted protein Populus trichocarpa	unknown function	-
HlMyb2	E3PA32	R2R3 Myb2 transcription factor Humulus lupulus	flavonoid biosynthesis	-
HlPTF	A2Q125	Putative transcription factor Humulus lupulus	unknown function	-
VvPUP-2	A5AZR6	Putative uncharacterized protein Vitis vinifera	unknown function	Velasco et al. 2007
GMYB9A	Q70RD1	MYB9A protein Gerbera hybrida	anthocyanin biosynthesis	Elomaa et al. 2003
EgMyb92	B3TLL1	MYB transcription factor MYB92 Elaeis guineensis var. Tenera	unknown function	Low et al. 2008
SsMyb3	A5A383	Myb transcription factor Solenostemon scutellarioides	anthocyanin biosynthesis	Nguyena et al. 2009
SmPAP1	D1MMA3	Transcription factor PAP1 Salvia miltiorrhiza	flavonoid biosynthesis	Wenping et al. 2011
AtMyb23	Q96276	transcription factor MYB23	trichome morphogenesis	Kirik et al. 2005
HlMyb3	A7WMF5	Myb3 factor Humulus lupulus	flavonoid biosynthesis	Matoušek et al. 2007
CsMyb5	B6DQ66	Myb-like transcription factor Myb5 Cucumis sativa	unknown function	Xia et al. 2009
VvMYB108	B9VSG9	R2R3 transcription factor MYB108-like protein 2	unknown function	Matus et al. 2008
VvPUP-1	D7U2M6	Putative uncharacterized protein Vitis vinifera	unknown function	-
VvPUP	A5C1Y6	Putative uncharacterized protein Vitis vinifera	unknown function	Velasco et al. 2007
GkMYB5	Q8H257	Myb-like transcription factor 5 Gossypioides kirkii	unknown function	Cedroni et al. 2003
GhMYB	O49020	Myb-like DNA-binding domain protein	unknown function	Loguerico et al. 1999
GhMyb5-1	Q94JN5	Myb-like transcription factor 5 Gossypium hirsutum	unknown function	-
GrMyb2	Q8H259	Myb-like transcription factor 2 Gossypium raimondii	unknown function	Cedroni et al. 2003
GhMyb5	Q8H260	Myb-like transcription factor 5 Gossypium hirsutum	unknown function	Cedroni et al. 2003
SbiMyb63	Q8S3Y6	Typical P-type R2R3 Myb protein Sorghum bicolor	unknown function	Jiang 2004
HlMyb6	FR873649	myb transcription factor 6 Humulus lupulus	unknown function	-
DcMyb3-2	A4GZI3	Transcription factor DcMYB3-2 Daucus carota	flavonoid biosynthesis	Wako et al. 2010

VvMybPA2	B8RCA6	MybPA2 Vitis vinifera	proanthocyanidin biosynthesis	Terrier et al. 2009
DkMyb2	D0VYJ7	Putative MYB transcription factor Diospyros kaki	proanthocyanidin biosynthesis	Akagi et al. 2009
AtMyb123TT2	Q9FJA2	transcription factor AtMyb123	flavonoid biosynthesis	Nesi et al. 2001
LjTT2a	B1B3F2	R2R3-MYB transcription factor Lotus japonicus	flavonoid biosynthesis	Yoshida et al. 2008
ZmC1	P10290	Zea mays C1	anthocyanin biosynthesis	Paz-Ares et al. 1987
AtMyb75PAP	Q9FE25	anthocyanin pigment 1 protein	anthocyanin biosynthesis	Borevitz et al. 2000
PiAN2	Q9M720	Petunia integrifolia	anthocyanin biosynthesis	Quattrocchio et al. 1999
InMYB1	Q1JV09	R2R3-MYB transcriptional regulator Ipomea nil	anthocyanin biosynthesis	Morita et al. 2006
InMYB2	Q1HAY2	R2R3-MYB transcriptional regulator Ipomea nil	anthocyanin biosynthesis	Morita et al. 2006
InMYB3	Q1HAY1	R2R3-MYB transcriptional regulator Ipomea nil	anthocyanin biosynthesis	Morita et al. 2006

*** References related to this table:**

Akagi T. et al. (2009) Plant Physiol. 151, 2028-2045.
Banks J.A. et al.(2011) Science 332, 960-963.
Borevitz J.O. et al. (2000) Plant Cell 12:2383-2393.
Cedroni,M.L. (2003) Plant Mol. Biol. 51 (3), 313-325.
Elomaa P. et al. (2003) Plant Physiol. 133:1831-1842.
Grotewold E. et al. (1991) Proc. Natl. Acad. Sci. U.S.A. 88, 4587-4591.
Jiang C. et al. (2004) Gene 326,13-22.
Kirik V. et al. (2005) Development 132,1477-1485.
Loguerico L.L. et al. (1999) Mol Gen Genet. 261(4-5), 660-71.
Low E.T. et al. (2008) BMC Plant Biol. 8, 62-62.
Matoušek, J. et al. (2007) J. Agric. Food Chem. 55 (19), 7767-7776.
Matoušek, J. (2005) J. Agric. Food Chem., 53 (12), 4793-4798.
Matus J.T. et al. (2008) BMC Plant Biol. 8, 83-83.
Mehrtens F. et al. (2005) Plant Physiol. 138,1083-1096.
Morita Y. et al. (2006) Plant Cell Physiol. 47:457-470.
Nesi N. et al. (2001) Plant Cell 13, 2099-2114.
Nguyena P. and Dal Cin V. (2009) Plant Phys. Biochem. 47(10), 934-945.
Paz-Ares J. et al. (1987) EMBO J. 6,3553-3558.
Quattrocchio F. et al. (1999) Plant Cell 11,1433-1444.
Terrier N. (2009) Plant Physiol. 149(2),1028-1041.
Velasco R. et al. (2007) PLoS ONE 2:e1326-e1326.
Wako T. et al. (2010) Plant Biotechnol. 27(2), 131-139.
Wenping H. et al. (2011) Genomics, 98(4), 272-279.
Yoshida et al. (2008) Plant Cell Physiol, 49(2), 157-169.
Xia X.J. et al. (2009) Plant Physiol. 150,801-814.

Additional file 2

B - Alignment of the R2R3 Myb domains of TFs compared to novel cloned hop Mybs (underlined):

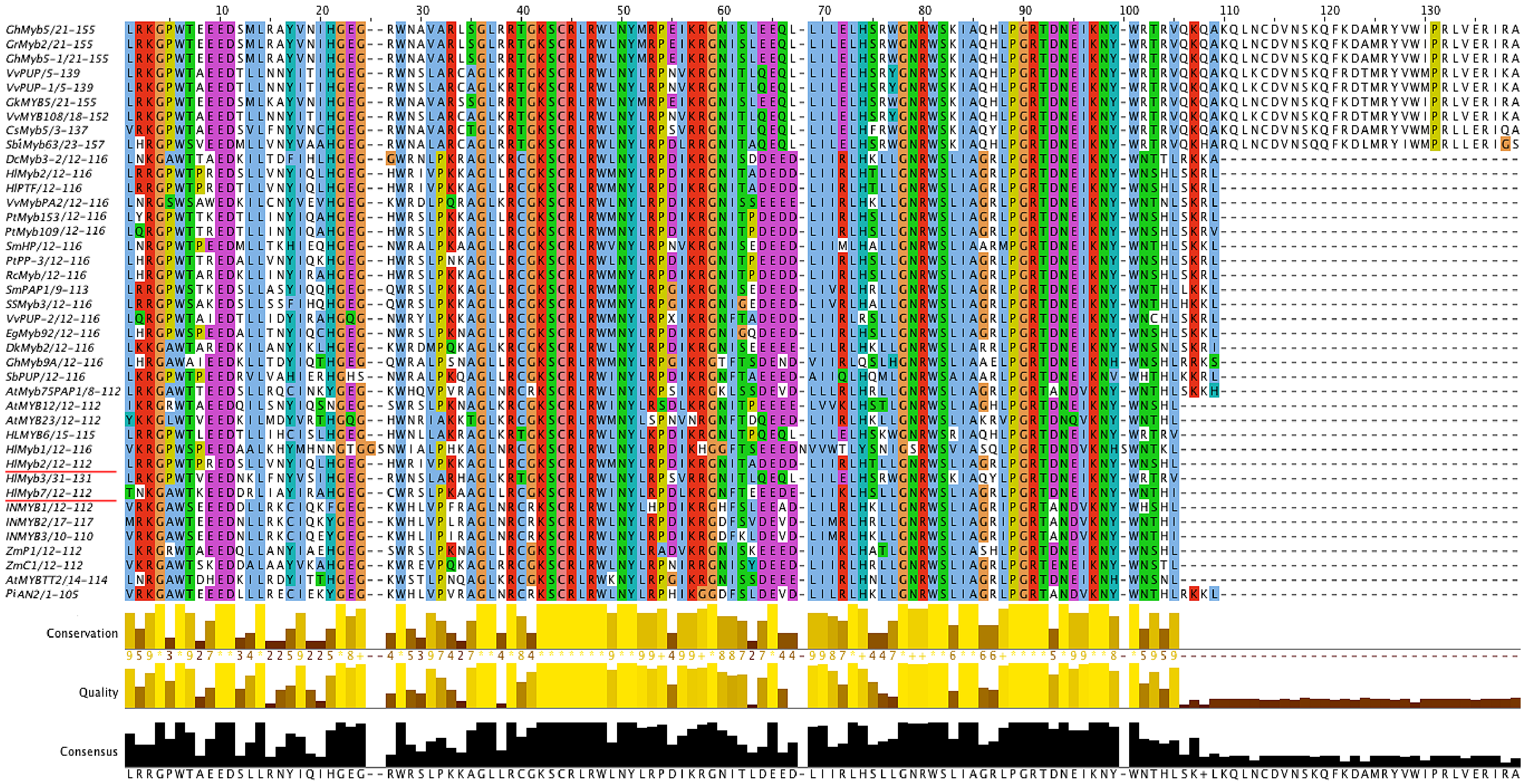

Supplement: Additional file 1 — List of R2R3Myb TFs included in the phylogenetic tree presented in Figure 1 and alignment of amino acid sequences within R2R3 domain. [file 1471-2229-12-27-S1.PDF]
