# Supplementary material for: Combinatorial analysis of lupulin gland transcription factors from R2R3Myb, bHLH and WDR families indicates a complex regulation of chs_H1 genes essential for prenylflavonoid biosynthesis in hop (Humulus Lupulus L.)
Source: BMC Plant Biol. 2012 Feb 20;12:27. doi: 10.1186/1471-2229-12-27 (PMC3340318; doi:10.1186/1471-2229-12-27)
Supplement: Additional file 2 — Comparative analysis of the 3-D protein structures of the N-terminal/R2/R3 regions of HlMyb2, l-HlMyb3 and s-HlMyb3. The theoretical structures were calculated and portrayed against the template 1h88C.pdb using the SWISS-MODEL Workspace. The alignments of the 3-D structures were performed using the Swiss-PdbViewer v3.7b2 (see Methods). Structures are presented as single ribbons, positions of R2 and R3 repeats are shown on alignment s-HlMyb3/l-HlMyb3; the positions of essential residues forming the bHLH-binding site of HlMyb2 are shown on the structure alignment for s-HlMyb3/HlMyb2. The positions of hydrophobic amino acids substituting the bHLH-binding residues in s-HlMyb3 are between brackets. The positions of the largest structural deviations are indicated on the structures by filled and hollow arrows. N and C are N- and C- terminus, respectively. [file 1471-2229-12-27-S2.PDF]

Figure S1 - Comparative analysis of the 3-D protein structures of the N-terminal/R2/R3 regions of *Hl*Myb2, l-*Hl*Myb3 and s-*Hl*Myb3.

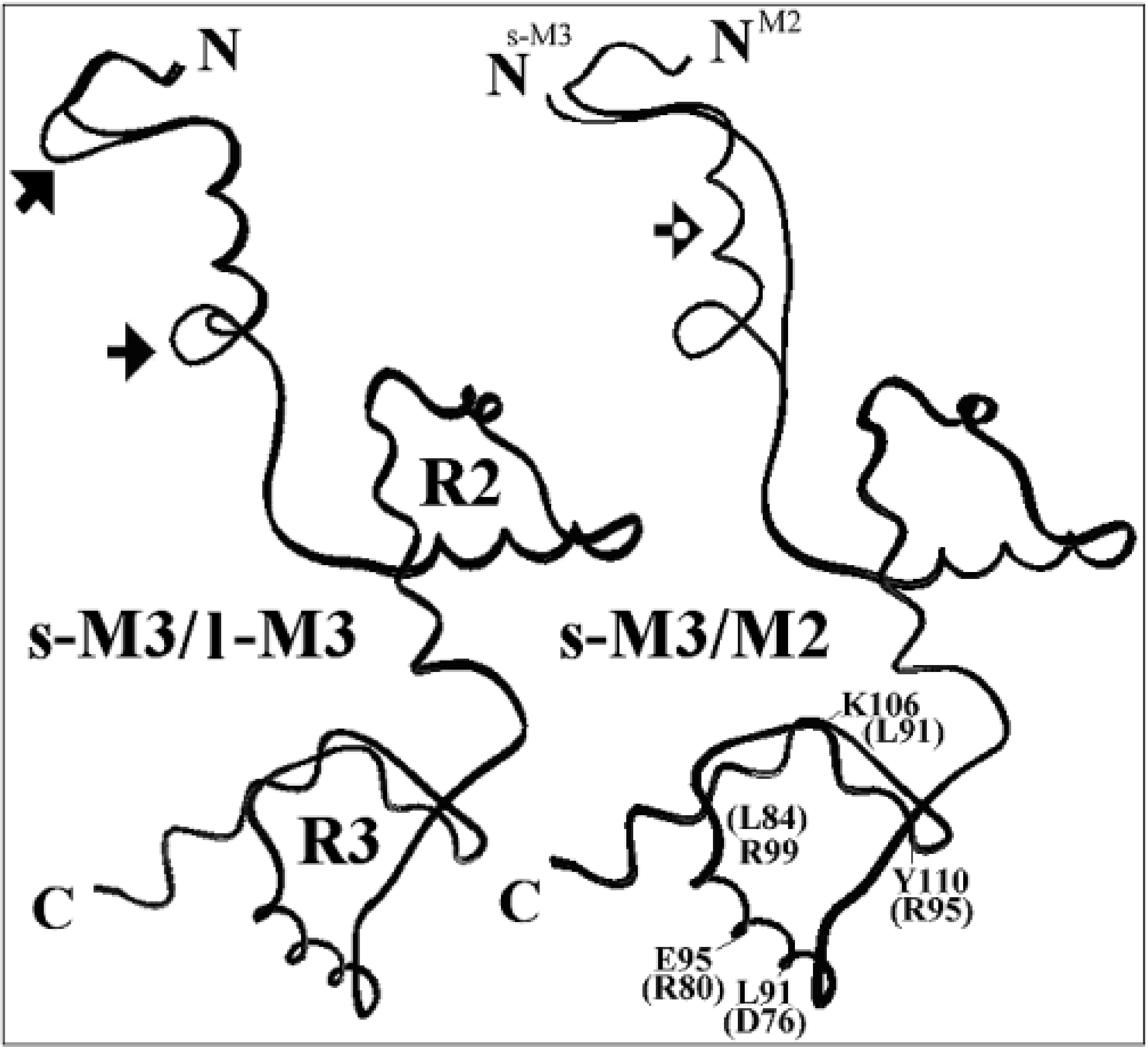

The theoretical structures were calculated and portrayed against the template 1h88C.pdb using the SWISS-MODEL Workspace. The alignments of the 3-D structures were performed using the Swiss-PdbViewer v3.7b2 (see Methods). Structures are presented as single ribbons, positions of R2 and R3 repeats are shown on alignment s-*Hl*Myb3/l-*Hl*Myb3; the positions of essential residues forming the bHLH-binding site of *Hl*Myb2 are shown on the structure alignment for s-*Hl*Myb3/*Hl*Myb2. The positions of hydrophobic amino acids substituting the bHLH-binding residues in s-*Hl*Myb3 are between brackets. The positions of the largest structural deviations are indicated on the structures by filled and hollow arrows. N and C are N- and C- terminus, respectively.
