# Supplementary material for: Combinatorial analysis of lupulin gland transcription factors from R2R3Myb, bHLH and WDR families indicates a complex regulation of chs_H1 genes essential for prenylflavonoid biosynthesis in hop (Humulus Lupulus L.)
Source: BMC Plant Biol. 2012 Feb 20;12:27. doi: 10.1186/1471-2229-12-27 (PMC3340318; doi:10.1186/1471-2229-12-27)

A - List of bHLH TFs included in the phylogenetic tree presented in Figure 2A

TF name	Other names	GenBank, Locus*	Swiss-Prot accession no.	Description	Proposed Biological Function	Reference**
VvMyc1	-	ACC68685.1	B7SNG3	BHLH-like DNA binding protein Vitis vinifera	anthocyanin biosynthesis	Hichri et al. 2010
PtPP	-	XP_002302118.1	B9GSP8	Predicted protein Populus trichocarpa	unknown function	-
RcCHP	-	XP_002520758.1	B9S439	Conserved hypothetical protein Ricinus communis	unknown function	-
NtAn1a	-	AEE99257.1	F6KRH4	Anthocyanin 1a Nicotiana tabacum	anthocyanin biosynthesis	Bai et al. 2011
PhAn1	-	AAG25927.1	Q9FEA1	Anthocyanin 1 Petunia hybrida	anthocyanin biosynthesis	Spelt et al. 2000
HlbHLH2	-	FR751553		bHLH TF from Humulus lupulus		-
MdbHLH3	-	AEI84807.1	D9ZIP4	bHLH transcription factor Malus domestica	anthocyanin biosynthesis	-
DvIVS	-	BAJ33515.1	E3WH98	bHLH transcriptional factor Dahlia pinnata	anthocyanin biosynthesis	-
PsbHLH	-	ADO13282.1	E3SXU4	bHLH Pisum sativum	unknown function	-
LjTT8	-	BAH28881.1	C0A1H0	bHLH transcription factor LjTT8 Lotus japonicus	flavonoid biosynthesis	Yoshida et al. 2010
LhbHLH2	-	BAE20058.1	Q401N4	bHLH transcription factor Lilium hybrid division I	anthocyanin biosynthesis	Nakatsuka et al. 2009
InbHLH2	-	BAE94394.1	Q1JV07	bHLH transcriptional factor Ipomoea nil	anthocyanin biosynthesis	Morita et al. 2006
IcbHLH2	-	ABY26932.1	A9YF25	putative anthocyanin transcriptional regulator Ipomoea coccinea	anthocyanin biosynthesis	Streisfeld & Rausher 2007
OsRc	-	BAF42668.1	A1IHC8	bHLH protein Oryza sativa Indica Group	proanthocyanidin synthesis	Furukawa et al. 2007
OsBPSC	-	ABB17166.1	Q2I7J3	Brown pericarp and seed coat Oryza sativa	anthocyanin biosynthesis	Sweeney et al. 2006
PfF3G1	-	BAC56998.1	Q852P3	F3G1 Perilla frutescens	anthocyanin biosynthesis	-
PiceabHLH	PsU	ABR17818.1	B8LQ88	Unknown Picea sitchensis	unknown function	-
OsbHLH009	ABB48017.1	LOC_Os10g42430	Q336P5	transcription factor MYC7E,putative, Oryza sativa Japonica Grp.	unknown function	-
AtbHLH004	MYC4	At4g17880	O49687	MYC4 JAZ-interacting transcription factor	anthocyanin biosynthesis	-
AtbHLH021	MS	At2g16910	Q9ZVX2	AMS Helix-loop-helix transcription factor	pollen development	Sorensen et al. 2003
AtbHLH029	FRU	At2g28160	Q0V7X4	FE-deficiency induced transcription factor 1	iron uptake responses	Jakoby et al. 2004
AtbHLH116	ICE1	At3g26744	Q9LSE2	Inducer of CBP expression 1, protein ubiquitination	freezing tolerance	Chinnusamy et al. 2004
AtbHLH033	SCRM	At1g12860	Q9LPW3	Inducer of CBF expression 2	stomatal differentiation	Kanaoka et al. 2008
AtbHLH022	DYT1	At4g21330	O81900	bHLH transcription factor expressed in tapetum	pollen development	Zhang et al. 2006
AtbHLH045	MUTE	At3g06120	Q9M8K6	bHLH TF contr. meristemoid differentiation in stomatal dev.	stomatal differentiation	Pillitteri et al. 2007
AtbHLH042	TT8	At4g09820	Q9FT81	TT8 regulation factor	proanthocyanidin synthesis	Nesi et al. 2000
AtbHLH020	NAI1	At2g22770	Q8S3F1	regulates development of ER bodies	formation of ER bodies	Matsushima et al. 2004
AtbHLH105	ILR3	At5g54680	Q9FH37	Iaa-leucine resistant 3 transcription factor	resistance to IAA conjugates	-
AtbHLH012	MYC1	At4g00480	C0SVG1	MYC related bHLH transcription factor	trichome formation	Symonds et al. 2011
AtbHLH001	GL3	NP_680372.1	Q9FN69	transcription factor GLABRA 3 Arabidopsis thaliana	trichome formation	Payne et al. 2000
AtbHLH002	EGL1	At1g63650	Q9CAD0	Enhancer of GLABRA3	trichome formation /anthocyanins	Bernhardt et al. 2003
HlbHLH1		CBI83256.1	E3PA31	bHLH TF from Humulus lupulus	unknown function	-
AtbHLH102	BIM2	At1g69010	Q9CAA4	BES1-interacting Myc-like protein 2	dTDP-rhamnose biosynthetic process	-

AtbHLH095	RGE1	At1g49770	Q9FXA3	Protein RETARDED GROWTH OF EMBRYO 1	embryo development	Kondou et al. 2008
AtbHLH039	ORG3	At3g56980	Q9M1K0	OPB3 responsive gene	iron stress	Kang et al. 2003
AtbHLH136	PRE1	At5g39860	Q9FLE9	Paclobutrazol 1 resistance	photomorphogenesis	-
AtbHLH159	P1R2	At4g30410	Q9M0B9	A.thaliana bHLH protein	unknown function	-
AtbHLH168	P1R3	At3g29370	Q8LD48	A.thaliana bHLH putative protein	unknown function	-
AtbHLH155	CPUORF7	At2g31280	Q9SJW8	conserved peptide upstream open reading frame 7	unknown function	-
AtbHLH083	RHD6	At1g66470	Q9C707	Root hair defective 6	root hair formation	Singh et al. 2008
OsbHLH123	OsLAX	LOC_Os01g61480	Q7XAQ6	Transcription factor LAX PANICLE Oryza sativa Japonica Grp.	shoot branching	Komatsu et al. 2003
AtbHLH154	ERP	At2g31730	Q9SKC7	A.thaliana bHLH protein	unknown function	-
AtbHLH059	UNE12	At4g02590	B9DFF4	unfertilized embryo sac 12	embryo development	-
AtbHLH108	MEE8	At1g25310	Q9FRI0	maternal effect embryo arrest 8	embryo development	-
AtbHLH050	BEE8	At1g73830	Q8GWK7	BR enhanced expression, positive regulator of BR signaling	brassinosteroid signaling	-
AtbHLH142	SAC51	At5g64340	Q9FMF4	bHLH transcription factor for SAC51, Protein suppressor of ACAULIS 51	plant morphogenesis	-
AtbHLH073	ALC	At5g67110	Q9FHA2	Myc/bHLH transcription factor like protein	fruit dehiscence	Rajani, Sundaresan 2001
AtbHLH024	SPT	At4g36930	Q9FUA4	transcription factor SPATULA	floral organogenesis	Alvarez, Smyth, 1999
AtbHLH016	UNE10	At4g00050	Q8GZ38	unfertilized embryo sac 10	embryo development	-
AtbHLH07	2PIF7	At5g61270	Q570R7	bHLH phytochrome interacting factor	light-signal transduction	Leivar et al. 2008
OsbHLH102	Os12g0610200	Os12g41650	Q0IM00	HLH DNA-binding domain cont. protein, Oryza sativa Japonica Grp.	unknown function	-
AtbHLH015	PIF1	At2g20180	Q8GZM7	phytochrome interacting factor like 3	light-signal transduction	Shen et al. 2005
AtbHLH065	PIF5	At3g59060	Q84LH8	member of PIF3 transcription factor family	light-signal transduction	Khanna et al. 2007
AtbHLH009	PIF4	At2g43010	Q8W2F3	member of PIF3 transcription factor family	light-signal transduction	Huq , Quail 2002
AtbHLH008	PIF3	At1g09530	O80536	TF acting positively in the phytochrome signaling pathway	light-signal transduction	Ni et al. 1998
AtbHLH026	HFR1	At1g02340	Q9FE22	light inducible nuclear bHLH	light-signal transduction	Fairchild et al. 2000
AtbHLH124	PIL1	At2g46970	Q8L5W8	phytochrome interacting factor	light-signal transduction	Salter et al. 2003
AtbHLH132	PIL2	At3g62090	Q8L5W7	phytochrome interacting factor	light-signal transduction	Salter et al. 2003

*** For Arabidopsis TFs, AGI codes were used**

**** References related to this table:**

Alvarez J., Smyth D.R. (1999) Development 126, 2377-2386.
Bai Y.et al. (2011) Planta 234(2):363-375.
Bernhardt C. (2003) Development 130, 6431-6439.
Fairchild C.D. et al. (2000) Genes Dev. 14, 2377-2391.
Furukawa T. et al. (2007) Plant J. 49, 91-102.

Hichri I. et al. (2010) Mol Plant. 3(3):509-523.
Huq E., Quail P.H. (2002) EMBO J. 21, 2441-2450.
Chinnusamy V. et al. (2003) Genes Dev. 17, 1043-1054.
Jakoby M. et al. (2004) FEBS Lett. 577, 528-534.
Kanaoka M.M. et al. (2008) Plant Cell 20, 1775-1785.
Kang H.-G. et al. (2003) Plant J. 35, 362-372.
Khanna R. et al. Plant Cell 19, 3915-3929.
Komatsu K. et al. (2003) PNAS 100, 11765-11770.
Kondou Y. et al. (2008) Plant Physiol. 147,1924-1935.
Leivar, P. et al. (2008) Plant Cell 20(2), 337-352.
Matsushima R. et al. (2004) Plant Cell 16,1536-1549.
Morita Y. et al. (2006) Plant Cell Physiol. 47:457-470.
Nakatsuka A.(2009) Sci. Hortic. 121, 84-91.
Nesi N. et al. (2000) Plant Cell 12, 1863-1878.
Ni M. et al. (1998) Cell 95, 657-667.
Payne C.T. et al. (2000) Genetics 156, 1349-1362.
Pillitteri L.J. et al. (2007)Nature 445, 501-505.
Rajani S., Sundaresan V. (2001) Curr. Biol. 11, 1914-1922.
Salter M.G. et al. Nature (2003) 426, 680-683.
Shen H. et al. (2005) Plant J. 44, 1023-1035.
Singh S.K. (2008) BMC Plant Biol., 16(8), 57.
Sorensen A.-M. et al. (2003) Plant J. 33, 413-423.
Spelt C. et al. (2000) Plant Cell 12, 1619-1632.
Streisfeld M.A., Rausher M.D.: (2007) Mol. Biol. Evol. 24:2816-2826.
Sweeney M.T. et al. (2006) Plant Cell 18, 283-294.
Symonds V.V. et al. (2011) PLoS Genet. 7:E1002069.
Yoshida K. et al. (2010) J. Plant Res. 123, 801-805.
Zhang W. et al. (2006) Development 133, 3085-3095.

B - Alignment of the bHLH domains of proteins compared to novel cloned hop *Hl*bHLH2 TF (underlined):

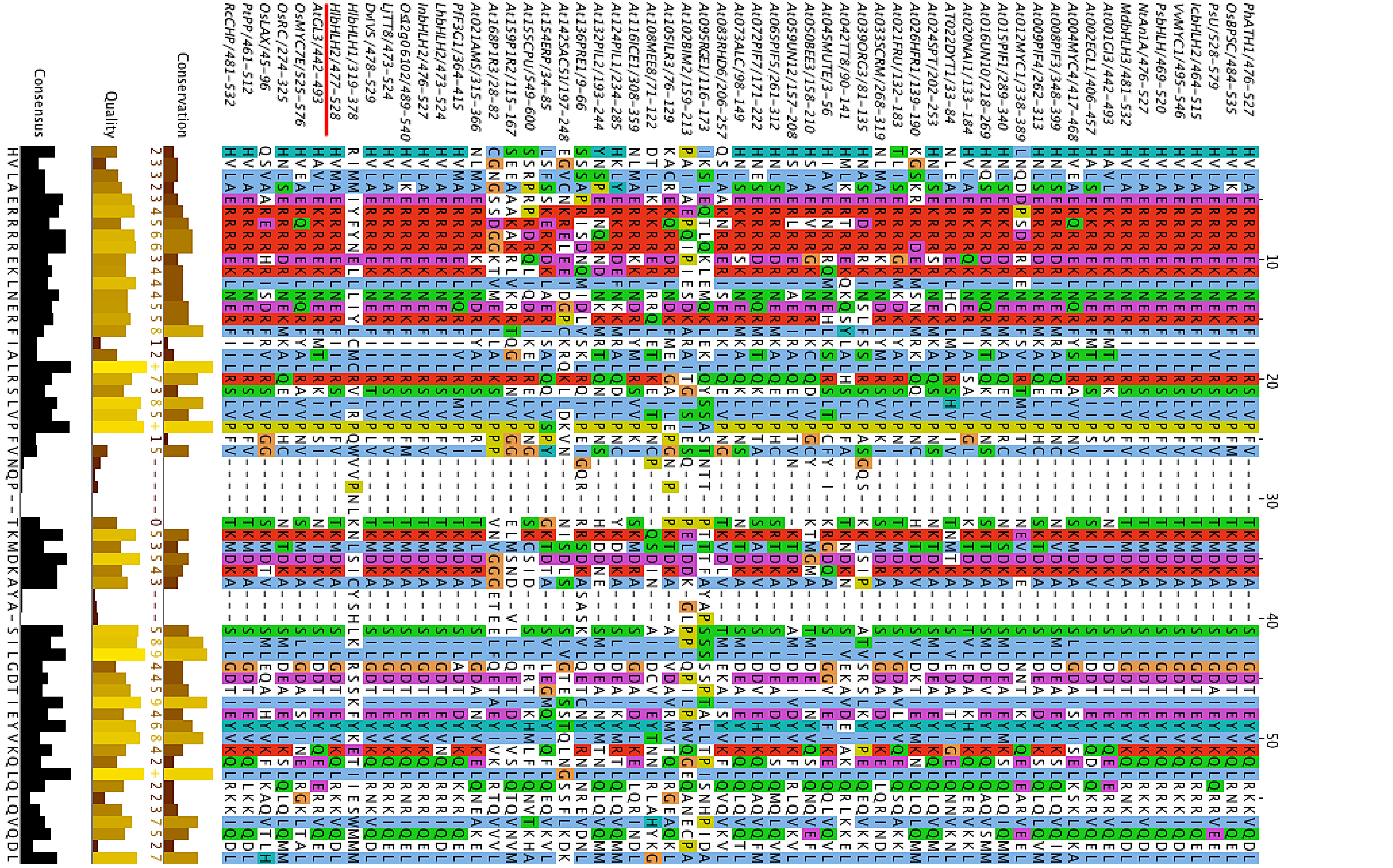

Supplement: Additional file 3 — List of bHLH TFs included in the phylogenetic tree presented in Figure 2A and alignment of amino acid sequences within bHLH domain. [file 1471-2229-12-27-S3.PDF]
