# Supplementary material for: Combinatorial analysis of lupulin gland transcription factors from R2R3Myb, bHLH and WDR families indicates a complex regulation of chs_H1 genes essential for prenylflavonoid biosynthesis in hop (Humulus Lupulus L.)
Source: BMC Plant Biol. 2012 Feb 20;12:27. doi: 10.1186/1471-2229-12-27 (PMC3340318; doi:10.1186/1471-2229-12-27)

A - List of WD40 TFs included in the phylogenetic tree presented in Figure 3A

TF name	Swiss-Prot accession no.	Description	Proposed Biological Function	Reference*
PgWD40	GenBank: ADV40946.1	WD40-repeat protein Punica granatum	anthocyanin biosynthesis	Ben-Simhon et al. 2011
MtWD40-1	B4XH97	WD40-1 protein Medico truncatula	anthocyanin biosynthesis	Pang et al. 2009
CjWDR	A5HMS8	transcription factor WD-repeat protein Caragana jubata	unknown function	-
PsA2	E5KXR1	A2 protein Pisum sativum	anthocyanin biosynthesis	Hellens et al. 2010
LjWD40	C0A1G9	WD40 repeats protein LjTTG1 Lotus japonicus	anthocyanin biosynthesis	Yoshida et al.2010
DvWDR1	E3WHA0	WD-repeats transcriptional factor Dahlia pinnata	anthocyanin biosynthesis	-
SmTTG1	C0LLW4	WD40 protein Saussurea medusa	unknown function	-
NtTTG1	C0M0P1	Transparent testa glabra 1-like protein Nicotiana tabacum	anthocyanin biosynthesis and trichome development	Wang et al. 2009
PhAN11	O24514	AN11 Petunia hybrida	anthocyanin biosynthesis	de Vetten et al. 1997
StAN11	F6KSJ4	AN11 Solanum tuberosum	anthocyanin biosynthesis	Zun-xi et al. 2008
InWDR1	Q1JUZ7	WDR1 Ipomoea nil	anthocyanin biosynthesis and trichome development	Morita et al. 2006
IhWD40	F1DPR0	WD40 repeat protein Ipomoea hochstetteri	anthocyanin biosynthesis	Streisfeld and Rauscher 2011
PyPWD40	E7EAX2	WD40 protein Pyrus pyrifolia	anthocyanin biosynthesis	Zhang et al. 2011
MdTTG1	Q9M610	TTG1-like protein Malus domestica	anthocyanin biosynthesis	Brueggemann et al. 2010
PpTTG1	C4NZQ9	Transparent testa glabra Prunus persica	unknown function	Taheri et al. 2011
HlWDR1	E3PQH4	WD-repeat protein Humulus lupulus	flavonoid biosynthesis	-
RbiTTG1	AEI55401	TTG1 protein Rubus idaeus	anthocyanin biosynthesis	Kassim et al. 2011
RcWD	B9SY15	WD-repeat protein, putative, Ricinus communis	unknown function	-
PotPP	B9I294	Predicted protein Populus trichocarpa	unknown function	-
GhTTG3	Q8LJT0	WD-repeat protein GhTTG3 Gossypium hirsutum	anthocyanin biosynthesis and trichome development	Humphries et al. 2005
CsTTG1	B7SBL6	WD-repeat protein Cucumis sativa	unknown function	-
AtTTG1	Q9XGN1	TTG1 Arabidopsis thaliana	anthocyanin biosynthesis and trichome development	Walker et al. 1999
BoWD40	E5D8F9	WD40 transcription regulator Brassica olearacea var. Botrytis	anthocyanin biosynthesis	Yuan et al. 2009
ZmPAC1	Q3V810	Anthocyanin byosynthetic gene regulator PAC1 Zea mays	anthocyanin biosynthesis	Carey et al. 2004
Picea	B8LLH5	putative uncharacterized WD40 protein Picea sitchensis	unknown function	-

*** References related to this table:**

Ben-Simhon Z. et al. (2011) Planta, *DOI: 10.1007/s00425-011-1438-4*
Brueggemann J. et al. (2010) Plant Cell Rep. 29(3), 285-294.

Carey,C.C. et al.: (2004) Plant Cell 16 (2), 450-464.
de Vetten,N. et al. (1997) Genes Dev. 11 (11), 1422-1434.
Hellens R.P. et al. (2010) PLoS ONE 5(10), e13230.
Humphries J.A. et al. (2005) Plant Mol. Biol. 57(1), 67-81.
Kassim, A. et al. (2009) Molec. Nutr. Food Res. 53: 625–634.
Zun-xi L. et al. (2008) Acta Hortic. Sinica, 35(9), 1317-1322.
Morita,Y. et al. (2006) Plant Cell Physiol. 47 (4), 457-470.
Streisfeld,M.A. et al. (2011) New Phytol. 191 (1), 264-274.
Taheri A. et al. (2011) In Vitro Cell. Dev. Biol. – Plant, *DOI: 10.1007/s11627-011-9390-3*
Walker A.R. et al. (1999) Plant Cell 11, 1337-1350.
Wang Y. et al. (2009) J. Cell Sci. 122(Pt 15), 2673-85.
Pang, Y. et al. (2009) Plant Physiol. 151,1114-1129.
Yoshida,K. et al. (2010) J. Plant Res. 123 (6), 801-805.
Yuan Y. et al. (2009) Planta 230(6), 1141-1153.
Zhang X. et al. (2011) Plant Mol. Biol. Rep. 29(2), 305-314.

B - Alignment of the WD40 repeat-containing proteins compared to novel cloned hop *Hl*WDR1 TF (underlined):

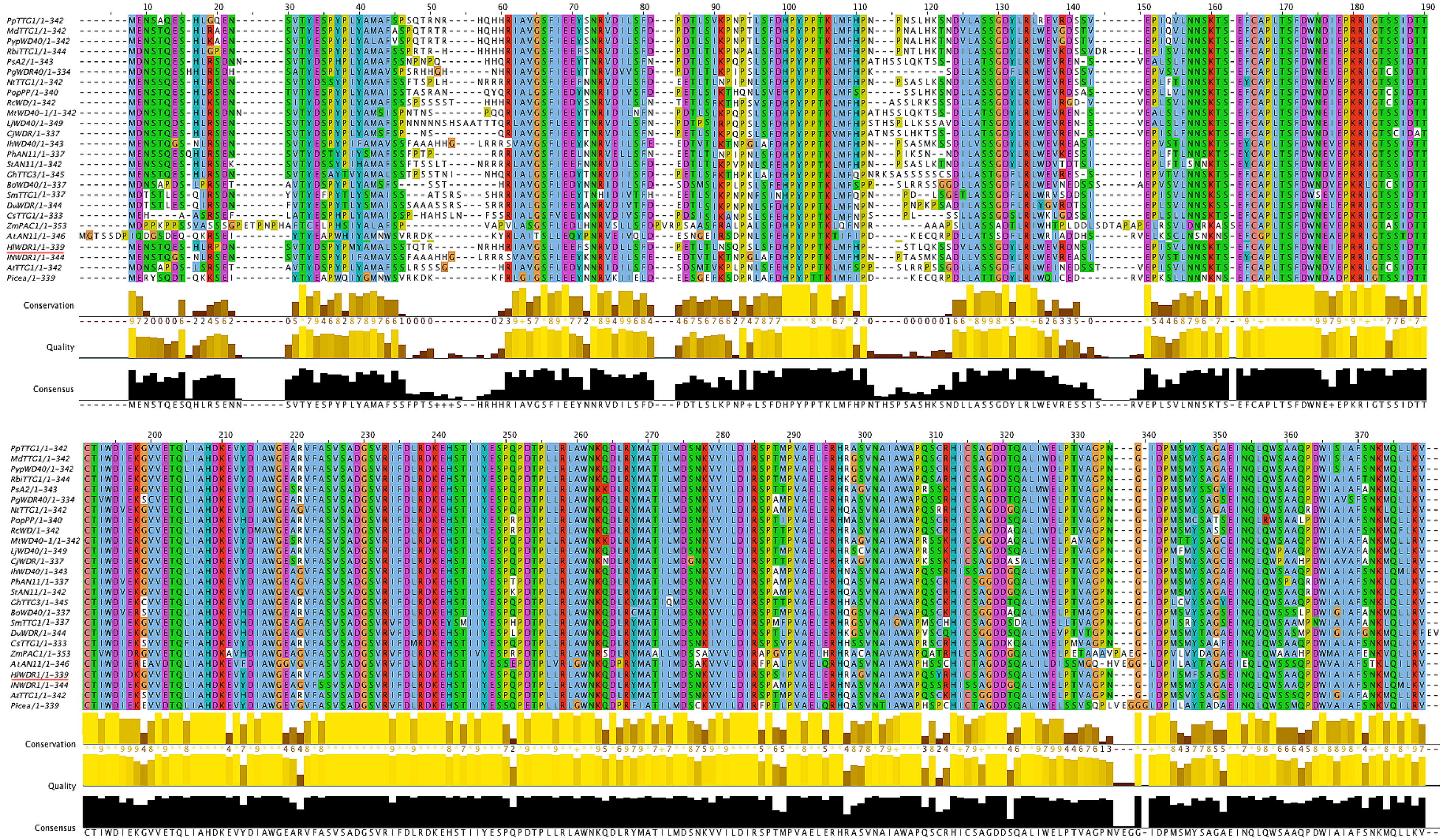

Supplement: Additional file 4 — List of WDR TFs included in the phylogenetic tree presented in Figure 3Aand alignment of amino acid sequences. [file 1471-2229-12-27-S4.PDF]
