# Supplementary material for: Combinatorial analysis of lupulin gland transcription factors from R2R3Myb, bHLH and WDR families indicates a complex regulation of chs_H1 genes essential for prenylflavonoid biosynthesis in hop (Humulus Lupulus L.)
Source: BMC Plant Biol. 2012 Feb 20;12:27. doi: 10.1186/1471-2229-12-27 (PMC3340318; doi:10.1186/1471-2229-12-27)
Supplement: Additional file 5 — Relative levels of anthocyanin pigments in petunia leaves (%) infiltrated with different hop TFs or AtPAP1 compared to anthocyanins in petunia dark blue corollas performed by HPLC analysis. [file 1471-2229-12-27-S5.PDF]

Relative levels of anthocyanin pigments# in petunia leaves (%) infiltrated with different hop TFs or *AtPAP1* compared to anthocyanins in petunia dark blue corollas*

Retention times (min)	Leaf samples/combinations					
	Control leaves	PAP1	B2	PAP1B2	PAP1B2W1	Flowers**
3.36	0	0	0	49.1	0	100
3.89	0	0	0	102.1	0	100
5.97	0	0	0	458.0	0	100
8.60	0	0	0	33.4	0	100
10.90	0	0	0	0.6	0	100
14.60	0	0	0	92.3	0	100
16.90	0	0	0	2.6	0	100
17.50	0	0	0	0	0	100
18.00	0	0	0	58.2	6.1	100
18.30	0	0	0	0	0	100
19.90	0	0	0	2.6	0.6	100

* No anthocyanin pigments were detected also in M2B2; s-M3B2; M2B2W1 and s-M3B2W1 combinations.
** Extract was made from dark blue flower corollas.

Note: Other secondary metabolites, mainly polyphenols were analyzed in the leaves of *P. hybrida* and *Nicotiana benthamiana* according to methods described previously (Matoušek *et al.* 2010). According to these results, no significant changes were detected except for complex combinations with *Hl*Myb3 TF. These spectra were specific for this TF as described previously (Matoušek *et al.* 2007, Matoušek *et al.* 2010).

Matoušek J, Vrba L, Novák P, Patzak J, De Keukeleire J, Škopek J, Heyerick A, Roldán-Ruiz I, De Keukeleire D: **Cloning and molecular analysis of the regulatory factor HlMyb1 in hop (Humulus lupulus L.) and the potential of hop to produce bioactive prenylated flavonoids.** J Agric Food Chem 2005, **53(12)**:4793-4798.

Matoušek J, Kocábek T, Patzak J, Stehlík J, Füssy Z, Krofta K, Heyerick A, Roldán-Ruiz I, Maloukh L, De Keukeleire D: **Cloning and molecular analysis of HlbZip1 and HlbZip2 transcription factors putatively involved in the regulation of the lupulin metabolome in hops (Humulus lupulus L.).** J Agric Food Chem 2010, **58(2):**902-912.
