# Supplementary material for: Combinatorial analysis of lupulin gland transcription factors from R2R3Myb, bHLH and WDR families indicates a complex regulation of chs_H1 genes essential for prenylflavonoid biosynthesis in hop (Humulus Lupulus L.)
Source: BMC Plant Biol. 2012 Feb 20;12:27. doi: 10.1186/1471-2229-12-27 (PMC3340318; doi:10.1186/1471-2229-12-27)
Supplement: Additional file 6 — Oligonucleotide primers used in this study. Table of oligonucleotide primers used in this study, arranged according the purpose for which they were designated. [file 1471-2229-12-27-S6.PDF]

List of oligonucleotide primers ordered according to the purpose of their use

**1) Primers for amplification of the genes from the lupulin gland-specific cDNA library**	
**Myb2 start**	5´ATGGGAAGAACAGCTTGTTG3´
**Myb2 stop**	5´TCAGCAAAAAGAA AAGTTCGATTG3´
**Myb7 start**	5´ATGGGAAGGTCTCCTTGTTG3´
**Myb7 stop**	5´CTATTTCATTTCCAAGCTTCTG3´
**bHLH start**	5´ATGTTGCAGACGGCGGTGC3´
**bHLH stop**	5´TCAACTATCACTATGCGGTATAATTTG3´
**WDR start**	5´ATGGAGAACTCGACGCAAG3´
**WDR snes**	5´ATGG AGAACTCGACGCAAGAATC3´
**WDR PCR anchor**	5’GACCACGCGTATCGATGTCGAC 3’
**Myb12start**	5’ATGGGAAGAGCGCCATGTTG3´
**Myb12stop**	5’TCATGACAGAAG CCAAGCGACC3´
**Myb23start**	5‘ATGAGA ATGACAAGAGATGGA3´
**Myb23stop**	5‘TCAAAGGCAATACCCATTAG3‘
**2) Primers for the real-time quantitative PCR**	
**HL-MYB2-F1**	5’TAGTGGGTCAGAGTACAGTGCTCAT3’
**HL-MYB2-R1**	5’CAACCTCGAGAAGCTGCTGATA3’
**HL-MYB7-F1**	5’ACCAACACGACCACCACAAT3’
**HL-MYB7-R1**	5’TGCGGATAAGGAGGGCTAAT3’
**HL-bHLH2-F1**	5‘AGCGGGTTGACTAGTGTGGAT3‘
**HL-bHLH2-R1**	5‘ACTTGCACCGTTGTCTCTGG3‘
**HL-WDR1-F1**	5‘TTGCTTAGATTGGCTTGGAATAA3‘
**HL-WDR1-R1**	5‘CCGGCTGAGCAGATATGTCTAT3‘
**HL-GAP-F1**	5‘ACCGGAGCCGACTTTGTTGTTG3‘
**HL-GAP-R1**	5‘TCGTACTCTGGCTTGTATTCCTTC3‘

**3) Primers for the cloning of the hop genes into plant expression vectors** (additional nucleotides to facilitate cleavage are indicated by small letters and restriction sites are underlined)	
**5’Myb2Apa**	5´GGGCCCATGGGAAGAACAGCTTGTTG 3´
**3’Myb2Kpn**	5´aaGGTACCTCAG CAAAAAGAAAAGTTCGATTG 3´
**5‘MYB7Xho**	5´taaCTCGAGATGGGAA GGTCT CCTTGTTG 3´
**5‘MYB7Xba**	5´taaTCTAGACTATTTCATTTCCAAGCT TCTG 3´
**bHLHKpn**	5´ taa GGTACCATGTTGCAGACGGCGGTG3´
**bHLHBam**	5´attGGATCCTCAACTATCACTATGCGGTAT3´
**5’HlWDXho**	5´aaCTCGAGGGCC CATGGAGAACTCGACGCAAG3´
**3’HlWDXba**	5´atTCTAGATCAAACTTTCAAAA GCTGC3´
**3’Myb12Xba**	5´atTCTAGATCATGACAGAAGCCAAGCGACC3´
**5‘Myb12Xho**	5´aaCTCGAGGGCCCATGGGAAGAGCGCCATGTTG3´
**5’Myb23Xho**	5’aaCTCGAGGGCCCATGAGAATGACAAGAGATGGA3´
**3’Myb23Xba**	5’aaTCTAGA T CAAAGGCAATACCCATTAG3´
**4) Primers for the preparation of chs_H1 promoter subvariants** (additional nucleotides to facilitate cleavage are indicated in the primer sequences by small letters and restriction sites are underlined)	
**1PchsH1Eco**	5’aaGAATTCGATCACGACCGTCCATTC3‘
**2PchsH1Eco**	5‘aaGAATTCCGCTTGTGTTGATGAGTTG3‘
**3PchsH1Eco**	5‘aaG AATTCCAACGAGCGCACCAAAC3‘
**4PchsH1Eco**	5’aaGAATTCGGCTTGGACTTAAGGTAGTT3‘
**5PchsH1Eco**	5’aaGAATTCTTGGCCAAGCACGTGAC3‘
**6PchsH1Eco**	5‘aaGAATTCAGCTACCCTTTTTTTTTTTAA3‘
**mutPmyb5**	5’aaGAATTCGATCACGACCGTCCATTCTTAACACCACAA ACGTAAGAAACG3‘ (mutated site double underlined)
**deltaPmyb5‘**	5‘aa GAATTCGATCACGACCGTCCATTCTTCCTACCACAAACGTAGCTACCCTTTTTTTTTTTAA3‘
**Pchs1endXba**	5‘aaTCTAGACATTTTTCCT TTAGTTTCGGA3‘
**Pchs4Eco**	5‘attGAATTCCGA TGA ACAACTAATAATAATATAAAAC3‘
**Pchs4Xba**	5‘AATTCTAGAAGTTACAGAT GCCATTACCTTACG3‘
