# Supplementary material for: Combinatorial analysis of lupulin gland transcription factors from R2R3Myb, bHLH and WDR families indicates a complex regulation of chs_H1 genes essential for prenylflavonoid biosynthesis in hop (Humulus Lupulus L.)
Source: BMC Plant Biol. 2012 Feb 20;12:27. doi: 10.1186/1471-2229-12-27 (PMC3340318; doi:10.1186/1471-2229-12-27)
Supplement: Additional file 7 — List of Agrobacterium tumefaciens strains and vectors. Table of A. tumefaciens strains and vectors used in the analysis of lupulin gland transcription factors from R2R3Myb, bHLH and WDR families. [file 1471-2229-12-27-S7.PDF]

List of *Agrobacterium tumefaciens* strains and vectors used in the analysis of lupulin gland transcription factors from R2R3Myb, bHLH and WDR families.

Agrobacterium strain: LBA4404
Plasmid vector: pLV-07*
Purpose: TF overexpression

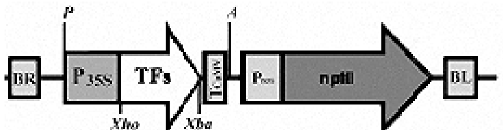

register no.	promoter /gene construct (TF)
1740	P35S::*At*PAP1
1773	P35S::s-*Hl*Myb3
1774	P35S::l-*Hl*Myb3
1789	P35S::*Hl*Myb1
2631	P35S::*Hl*bHLH1
2816	P35S::*Hl*Myb7
3074	P35S::*Hl*MYB2
3077	P35S::AtMYB23
3233	P35S::*Hl*WDR1
3236	P35S::*At*Myb12
3257	P35S::*At*TTG1
3577	P35S::*Hl*bHLH2

Agrobacterium strain: LBA4404
Plasmid vector: pBGF-0**
Purpose: transient expression, GUS assay

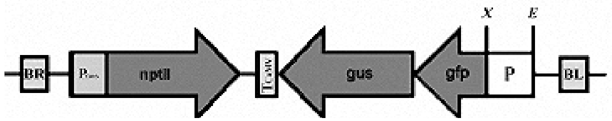

register no.	promoter (P)/gene construct
1006	original pBGF-0 construct**
3384	P*Hl*chs4::gfp/gus
3612	P2 *Hl*chs_H1:: gfp/gus
3613	P3 *Hl*chs_H1:: gfp/gus
3614	P4 *Hl*chs_H1:: gfp/gus
3615	P5 *Hl*chs_H1:: gfp/gus
3616	P6 *Hl*chs_H1:: gfp/gus
3652	MUT P*Hl*chs_H1:: gfp/gus
3653	ΔP*Hl*chs_H1:: gfp/gus
3771	P35:: gfp/gus

Agrobacterium strain: LBA4404
Plasmid vector: pLV-062
Purpose: real-time qPCR

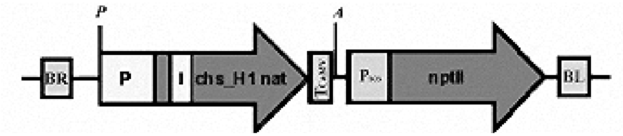

register no.	promoter (P)/gene construct
1741	chs_H1 including its native promoter

* Vrba L, Matoušek J (2005) Expression of modified 7SL RNA gene in transgenic Solanum tuberosum plants. Biol. Plant 49:371–380

** Chytilova, E., Macas, J., and Galbraith, D.W. (1999) Green fluorescent protein targeted to the nucleus, a transgenic phenotype useful for studies in plant biology. Ann. Bot. 83, 645–654.
