# Supplementary material for: Combinatorial analysis of lupulin gland transcription factors from R2R3Myb, bHLH and WDR families indicates a complex regulation of chs_H1 genes essential for prenylflavonoid biosynthesis in hop (Humulus Lupulus L.)
Source: BMC Plant Biol. 2012 Feb 20;12:27. doi: 10.1186/1471-2229-12-27 (PMC3340318; doi:10.1186/1471-2229-12-27)
Supplement: Additional file 8 — Histochemical and quantitative analysis of GUS activity driven by modified variants of Pchs_H1 by hop TFs (supplement to Table 1). [file 1471-2229-12-27-S8.PDF]

Additional file 8

Histochemical and quantitative analysis of GUS activity driven by modified variants of Pchs_H1 by hop TFs (supplement to Table 1)

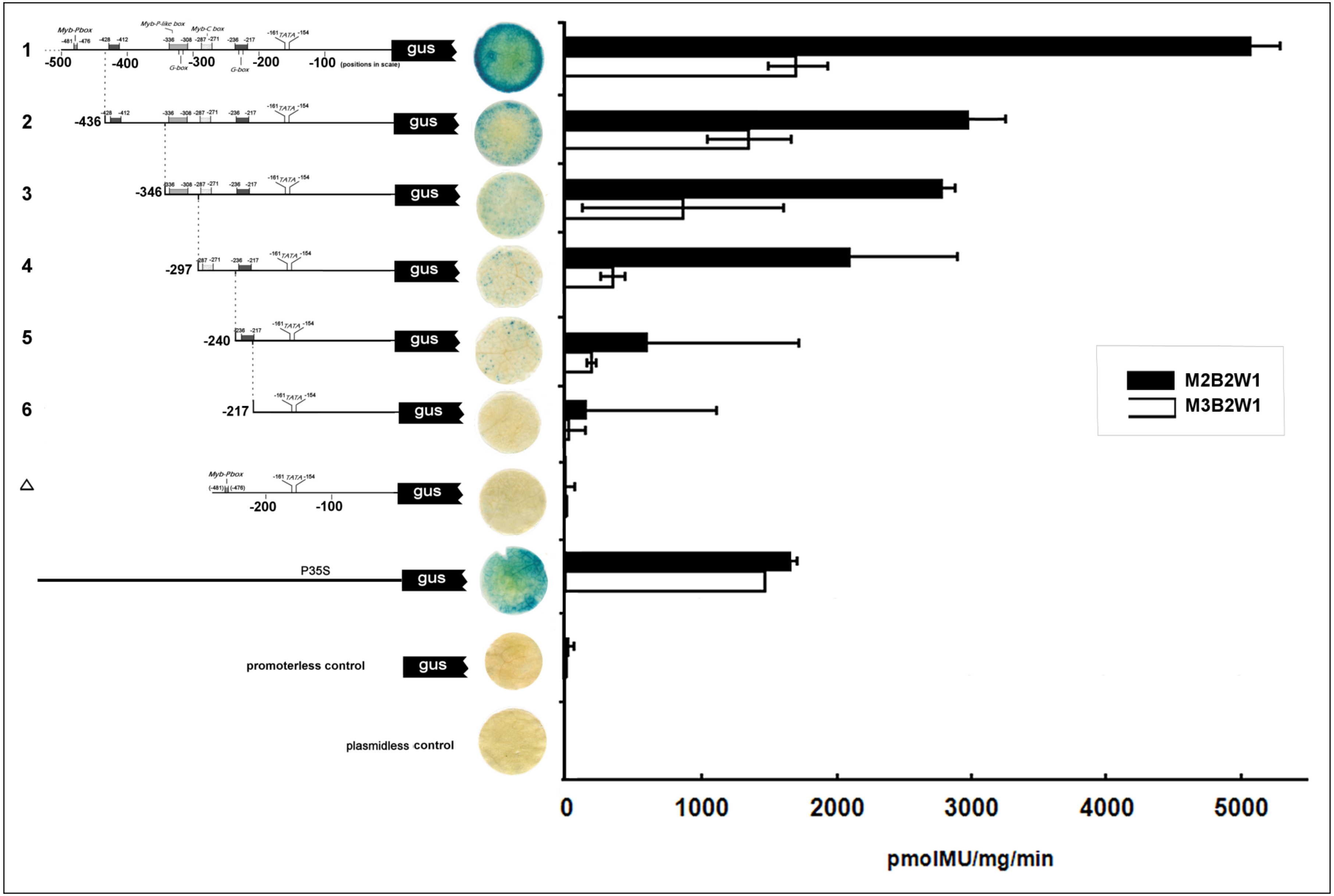

The leaf discs from infiltrated *N. benthamiana* plants were fixed in 0.3% formaldehyde and treated according to the Jefferson protocol*. Tissue samples were incubated in substrate (5-bromo-4-chloro-3-indolyl-glucuronide) solution overnight at 37°C. Removal of pigments was achieved by several washes in 50% to 70% (v/v) ethanol.

---

* Jefferson RA, Kavanagh TA, Bevan MW: **GUS fusions: beta-glucuronidase as a sensitive and versatile gene fusion marker in higher plants.** *EMBO J* 1987, **6**:3901–3907.
